# Optimizing homeostatic cell renewal in hierarchical tissues

**DOI:** 10.1371/journal.pcbi.1005967

**Published:** 2018-02-15

**Authors:** Cesar Alvarado, Nicole A. Fider, Helen J. Wearing, Natalia L. Komarova

**Affiliations:** 1 Department of Mathematics and Statistics, University of New Mexico, Albuquerque, New Mexico, United States of America; 2 Department of Mathematics, University of California Irvine, Irvine, California, United States of America; 3 Department of Biology, University of New Mexico, Albuquerque, New Mexico, United States of America; 4 Department of Ecology and Evolutionary Biology, University of California Irvine, Irvine, California, United States of America; Max-Planck-Institute for Evolutionary Biology, GERMANY

## Abstract

In order to maintain homeostasis, mature cells removed from the top compartment of hierarchical tissues have to be replenished by means of differentiation and self-renewal events happening in the more primitive compartments. As each cell division is associated with a risk of mutation, cell division patterns have to be optimized, in order to minimize or delay the risk of malignancy generation. Here we study this optimization problem, focusing on the role of division tree length, that is, the number of layers of cells activated in response to the loss of terminally differentiated cells, which is related to the balance between differentiation and self-renewal events in the compartments. Using both analytical methods and stochastic simulations in a metapopulation-style model, we find that shorter division trees are advantageous if the objective is to minimize the total number of one-hit mutants in the cell population. Longer division trees on the other hand minimize the accumulation of two-hit mutants, which is a more likely evolutionary goal given the key role played by tumor suppressor genes in cancer initiation. While division tree length is the most important property determining mutant accumulation, we also find that increasing the size of primitive compartments helps to delay two-hit mutant generation.

## Author summary

Cells in multicellular organisms are organized hierarchically. A stem cell gives rise to a chain of dividing and progressively differentiating offspring. At the end of this chain (called a lineage) are terminally differentiated cells that perform their function and undergo programmed cell death, to be replaced by new divisions of less differentiated cells. Here we are interested in the design of such lineages. At one extreme, one can imagine that a loss of terminally differentiated cells only results in divisions of cells in close hierarchical proximity to them, giving rise to very short division trees. On the other hand, it is possible that a long chain of increasingly primitive cells gets activated in response to the loss of differentiated cells. We expect that an important type of selection pressure acting upon tissue design is the minimization of mutations that happen in the course of everyday tissue maintenance (homeostasis). For example, tumor suppressor gene inactivation (two consecutive mutations) is an early rate-limiting step in many cancers. Using mathematical and computational methods, we find that the length of division trees is anti-correlated with the likelihood of double mutations, and lengthening the trees may provide an evolutionary advantage to the organism by delaying the onset of cancer.

## Introduction

Many tissues in complex organisms are hierarchically organized, such as the human colon, the small intestine, the skin, and the haematopoietic system. Hierarchically organized tissues are renewed constantly by means of a balance of cell deaths and cell divisions. Understanding the mechanisms that regulate cell self-renewal and differentiation patterns is key to explaining the robust nature of homeostasis [1]. Because of the imminent risk of mutation acquisition, tissue organization also plays an important role in preventing cancer [2–9].

At the root of hierarchically organized tissues are a small number of stem cells (SCs), capable of both self renewal and differentiation into more specialized cells [10]. Downstream from SCs there are intermediate cells of increasing degrees of maturity, which can undergo a certain number of divisions. Finally, there are fully mature (terminally) differentiated cells, which perform their function and are discarded and replenished through divisions of less differentiated cells. For instance, evidence suggests that on or near the bottom of the colon crypts and the small intestine crypts, there are between 4 − 6 SCs [11–13], and the remaining compartments are composed of transit amplifying (TA) cells and finally mature differentiated cells that are discarded, so that the entire crypt is renewed every 2 − 7 days [14]. A similar situation occurs in the haematopoietic system, where a stem cell pool of about 400 cells is required to replenish a daily bone marrow output of about 3.5 × 10^11^ cells [15, 16].

**Fig 1:**
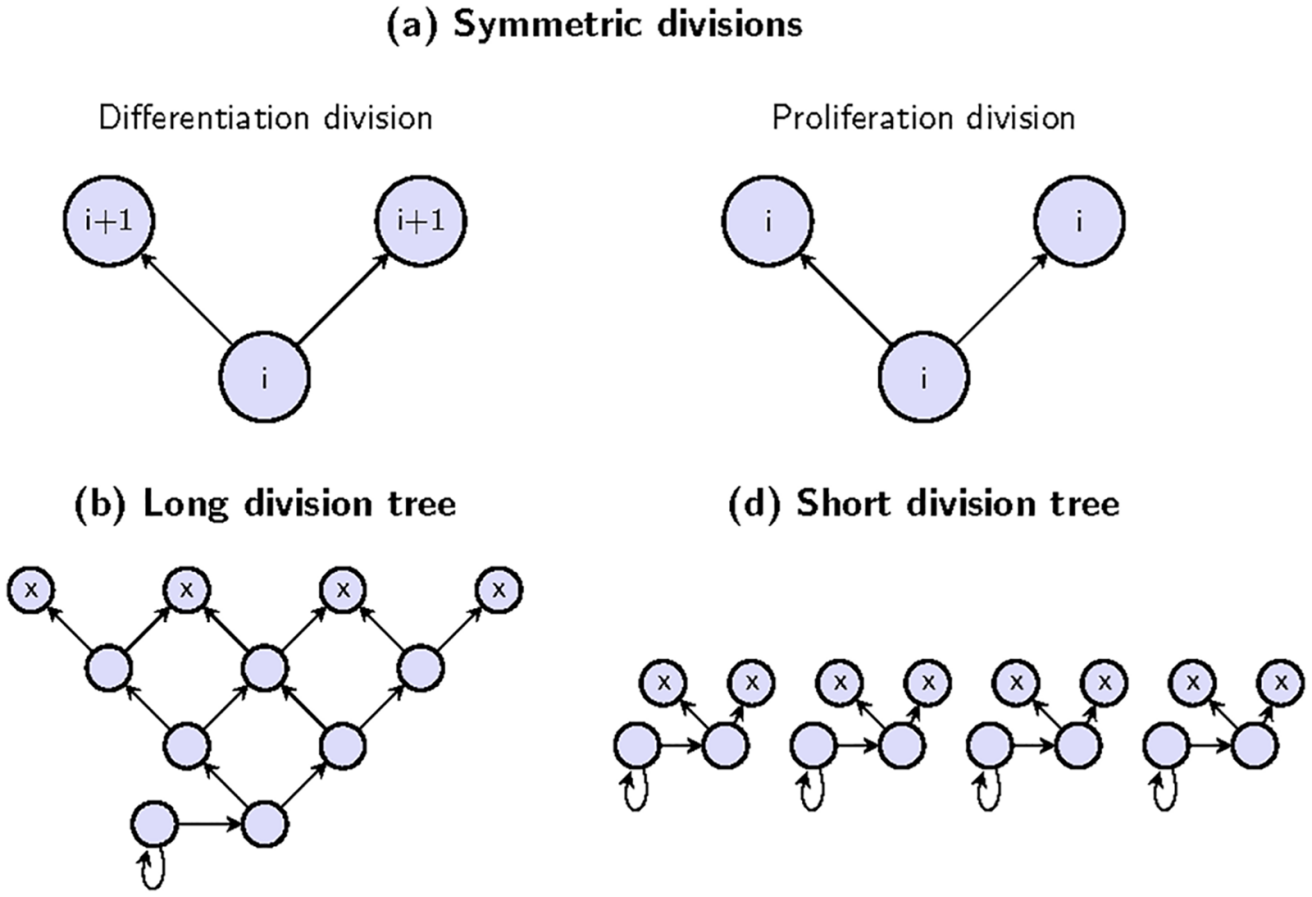
Schematics showing key concepts of the paper. (a) Two types of symmetric divisions: self-renewals and differentiations. Each circle represents a cell, and *i* denotes the *i*th compartment, while *i* + 1 denotes the (*i* + 1)th compartment. Panels (b) and (c) demonstrate the division chains that replenish 8 differentiated cells eliminated from the top compartment. Dead cells are denoted by X’s and arrows show divisions. Cells are arranged in horizontal layers corresponding to compartments. Only the dividing cells are shown (for example, there may be more than 4 cells in the second to top compartment in panel (b)). In (b), the dead cells are replaced by a longer division tree, and in (c) by four shorter division trees.

Stem cells are thought to be capable of symmetric or asymmetric divisions. Asymmetric divisions of a stem cell result in one daughter cell that is a stem cell and the other daughter cell that is a more differentiated cell. Symmetric divisions result in two identical daughter cells. The two types of symmetric divisions are illustrated in Fig 1(a) in a more general context: differentiation divisions result in two offspring that are both more differentiated than the dividing cell, and self-renewal divisions result in two offspring whose differentiation status is identical to that of the parent.

Because experiments to track cell divisions and mutations are in general difficult, or not feasible in some hierarchically organized tissues, mathematical models have been used to understand cellular dynamics or homeostasis and mutations. In particular, the origin and development of colorectal cancer have been extensively studied. It has been demonstrated theoretically that mutations leading to colorectal cancer can originate in either the stem cell compartment or TA cells [3, 5, 7, 17]. Computational models, such as virtual crypts, have helped to understand the process of self renewal in hierarchically organized tissues, for instance the organization of the colon [18–21]. Several studies have investigated tissue architecture with the goal of understanding its utility in protection against mutation accumulation. Traulsen, Werner and colleagues used mathematical models to study mutations in the haematopoietic system, and found theoretical evidence that tissue architecture and the process of self renewal were a protection mechanism against cancer [6, 9, 22, 23]. Rodriguez-Brenes et al. [8] proposed that an optimal tissue architecture that minimized the replication capacity of cells was one where the less differentiated cells had a larger rate of self-renewal. Another study [2] showed that having symmetric stem cell divisions (self-renewals and differentiations) rather than asymmetric stem cell divisions minimized the risk of two-hit mutant generation. Furthermore, Dingli et al. [24] considered the question of mutation generation by stem cells and found that mutations that increased the probability of asymmetric replication could lead to rapid expansion of mutant stem cells in the absence of a selective fitness advantage. Pepper et al. [25] examined a tissue undergoing serial differentiation patterns originating with self-renewing somatic stem cells, continuing with several TA cell differentiations, and showed that such patterns lowered the rate of somatic evolution. Finally, Sprouffske et al. [26] emphasized the importance of spatial considerations in the modeling of stem cell hierarchies and division patterns.

Despite significant progress reported in the literature, there are still unanswered questions regarding tissue renewal and cancer development in hierarchically organized tissues. In particular, the optimal mechanisms of self renewal and self-renewal to maintain homeostasis is a crucial process which is not completely understood. In a recent paper, [27] present an elegant model that allows one to calculate the optimal lineage structure that minimizes the divisional load of cells. The premise of this paper is that to limit the accumulation of somatic mutations, renewing tissues must minimize the number of times each cell divides during differentiation. On the other hand, as was discovered by Werner et al. in their analysis of mutant dynamics [23], the occurrence of a mutant and the compartment of origin and its subsequent clonal dynamics are all of importance. In the present study we consider an optimization problem, where the objective is to optimize observables that are important for cancer prevention/delay. Namely, our aim is to minimize the number of one-hit mutants accumulated in the tissue, and to maximize the expected time until two-hit mutants are generated.

We proceed by first formulating a top-down, hierarchical stochastic model of tissue self-renewal, and then deriving analytical expressions for the expected number of mutants in each compartment. This informs a deterministic approximation resulting in a set of differential equations describing mutant dynamics in different compartments. It turns out that this methodology can be further adapted to describe not only the approximately deterministic regime of large populations and large mutation rates, but a more relevant regime of small populations and small mutation rates. We investigate the dynamics of our model in different scenarios, focusing on different self-renewal/differentiation probabilities and different compartment size arrangements. In addition, we perform stochastic simulations to study the accumulation of mutations in a stochastic regime. We describe both one-hit and two-hit mutant generation, and find the parameters that can be tuned to delay cancer initiation in hierarchically organized tissues.

## Materials and methods

### The top-down approach

To illustrate the questions we are interested in solving, consider a hierarchical tissue where symmetric divisions are prevalent, such as the human colon [28–31]. When mature differentiated cells in the most downstream compartment (the “top” layer of tissue) are discarded, cells from the adjacent upstream compartment divide to replace the eliminated cells. This gives rise to a chain of differentiations, because the cells that differentiated and migrated from the upstream compartment also need to be replaced, in order to keep the tissue at homeostasis. Fig 1(b) and 1(c) schematically illustrates two opposite trends. Both panels depict 8 mature cells that are eliminated. These cells are replenished by 4 differentiation events, whereby cells from the neighboring compartment differentiate and migrate downstream to replace the dead cells. In panel (b), the 4 cells are, in turn, replenished by two differentiation events from the adjacent upstream layer (representing a compartment in the hierarchy), leaving two cells to be replaced. This is done by a single differentiation event from the bottom layer, and the remaining cell is then replaced by a self-renewal event from the same layer. These processes comprise a differentiation cascade, or a *differentiation chain*, which in this case includes differentiation events in 3 consecutive compartments. A different scenario is shown in panel (c) where the 4 cells that differentiate and migrate from the second-to-top layer are all replaced by self-renewal divisions of cells in the same layer. In this case we have 4 shorter differentiation chains which only include differentiation events in one compartment. As will be shown, a range of intermediate scenarios is possible.

Biologically, differentiation chains are shaped by a system of feedback loops determined by signals altering self-renewal, differentiation, apoptosis, migration, and adhesion in both the stem cell compartment and the transit amplifying cells [1, 8, 32]. The self-renewal and differentiation activity is a well controlled system to guarantee tissue homeostasis in hierarchically organized tissues, thus, changes in self-renewal and differentiation patterns might be associated with an increased risk of cancer development, as found, for example, by Merrit et al. [33] in the context of colorectal cancer. Fig 1(b) and 1(c) suggests that in terms of tissue architecture, and in the presence of hierarchical lineages, there are multiple arrangements that are all, in principle, capable of maintaining balanced tissue turnover. Each arrangement preserves the number of cells in each compartment in the face of divisions triggered by cell deaths in the top compartment. Because each cell division is associated with a probability of harmful mutations, the question then becomes if there is a preferred strategy for a tissue to arrange its turnover and/or compartment sizes in order to minimize the number of mutants generated and accumulated.

We note that in our top-down approach for the optimization problem, we assume that the number and the turnover rate of the terminally differentiated cells is a given constraint, dictated by the needs of the organ. It is the upstream arrangement of the less differentiated compartments that is under the evolutionary pressure to delay cancer. A precise formulation of this concept is presented next.

### The stochastic process

Assume that there are *n* + 1 compartments, *C*_0_, …, *C*_*n*_, with total constant cell numbers *N*_0_, …, *N*_*n*_. Inside individual compartments, we assume complete mixing, and the compartments are arranged linearly from the least mature, *C*_0_, to the most mature, *C*_*n*_. At each time step, 2^*n*^ cells from the most differentiated compartment *C*_*n*_ are removed. These cells must be replaced by 2^*n*^ divisions in different compartments.

This is done by going through the compartments consecutively in the upstream direction (*C*_*n*−1_, *C*_*n*−2_, …, *C*_0_) and probabilistically assigning the division event types that take place. The parameter that determines the division type is 0 ≤ *v*_*i*_ ≤ 1, which is the probability for a cell in compartment *C*_*i*_ to be replaced by means of a self-renewing division in the same compartment. We assume that *v*_*n*_ = 0, that is, terminally differentiated cells do not self-renew. Further, because there is no compartment below the stem cell layer, removed stem cells can only be replaced by self-renewal and *v*_0_ = 1 necessarily.

In the stochastic simulations, we let the size of each compartment *C*_*i*_ be constant (*N*_*i*_, for each *i* = 0, 1, …, *n*) and we track the numbers of mutants *m*_*i*_ in each of the compartments. The number of healthy cells is therefore *N*_*i*_ − *m*_*i*_, where each such cell can be thought of as a biological “wild card” in the sense that, if the cell is chosen for cell division, it will mutate with probability *u*.

The initial number of mutants is zero, and the simulation proceeds as a sequence of discrete updates. Each update starts with 2^*n*^ cells removed from compartment *C*_*n*_. A mutant is discarded with probability $\frac{m_{n}}{N_{n}}$, otherwise, the cell discarded is a wild type cell. The next step is to replace the 2^*n*^ cells removed in compartment *C*_*n*_. Because cells in compartment *C*_*n*_ cannot proliferate, 2^*n*−1^ cells differentiate out from compartment *C*_*n*−1_. In each division, a mutant is chosen with probability $\frac{m_{n-1}}{N_{n-1}}$, which increases the number of mutants in compartment *C*_*n*_ by two while the number of mutants in compartment *C*_*n*−1_ decreases by one. If a wild type cell is chosen for differentiation in compartment *C*_*n*−1_, a de-novo mutant is produced with probability *u*, such that the number of mutants in *C*_*n*_ increases by one. The number of wild-type cells in *C*_*n*−1_ is decreased by one. There are altogether 2^*n*−1^ differentiations from *C*_*n*−1_ performed in this way.

Next, 2^*n*−1^ cells have to be replaced in compartment *C*_*n*−1_. Each of these cells can be replaced by a self-renewal event in *C*_*n*−1_ with probability *v*_*n*−1_. Cells that are not replaced by a self-renewal event are replaced by a differentiation event from compartment *C*_*n*−2_ with probability 1 − *v*_*n*−1_. If the number of cells to be replaced by differentiation events is odd, we add an extra self-renewal event in compartment *C*_*n*−1_ so that the number of remaining openings is even. If the division event is a self-renewal, a mutant is chosen with probability $\frac{m_{n-1}}{N_{n-1}}$ which increases the mutant population in compartment *C*_*n*−1_ by one. If the proliferating cell is a wild type cell, it produces a de-novo mutant with probability *u* such that the number of mutants in compartment *C*_*n*−1_ increases by one. If the wild type cell did not mutate or a mutant was not chosen for reproduction, the number of wild type cells in compartment *C*_*n*−1_ increases by one. If the event is a differentiation from compartment *C*_*n*−2_, mutants and wild type cells are updated in the same way as described above for differentiations from compartment *C*_*n*−1_.

After this, there are a number of cells “missing” from compartment *C*_*n*−2_ that have to be replaced in a similar way. Every time a cell is chosen for division from compartment *C*_*i*_, it is a mutant with probability $\frac{m_{i}}{N_{i}}$. The process of cell replacement proceeds until compartment *C*_0_ is reached. Cells “missing” from this compartment are replaced by self-renewal divisions in the same compartment with probability *v*_0_ = 1.

### Trees and their probabilities

Here we will build all possible division trees that can result from removing 2^*n*^ cells from compartment *C*_*n*_. Then, given the values *v*_*i*_, we will calculate the probability for each possible division tree.

Since cells in compartment *C*_*n*_ do not proliferate, following *a*_*n*_ ≡ 2^*n*^ removals from *C*_*n*_, there will be *a*_*n*−1_ = 2^*n*−1^ differentiation divisions in compartment *C*_*n*−1_. Next, *a*_*n*−1_ cells in this compartment must be replaced, either by differentiation divisions from compartment *C*_*n*−2_ or by self-renewal divisions from compartment *C*_*n*−1_. Suppose there are *a*_*n*−2_ differentiations and *a*_*n*−1_ − 2*a*_*n*−2_ self-renewals. Next, *a*_*n*−2_ cells from compartment *C*_*n*−2_ must be replaced. This process gives rise to a division tree, which is uniquely characterized by the numbers of differentiation divisions in each compartment:

$$\begin{array}{c} \{a_{0},\ldots ,a_{n-1}\}, \end{array}$$ (1)

where *a*_*n*−1_ = 2^*n*−1^ and

$$\begin{array}{c} 0\leq a_{i}\leq 2^{i},\;i=0,\ldots ,n-1. \end{array}$$

The number of self-renewals in compartment *C*_*i*_ is given by *a*_*i*_ − 2*a*_*i*−1_. The length of the tree is the number of compartments that have nonzero numbers of differentiation events, and it varies from 1 to *n*. In other words, the length of the tree is the number of cell levels that activated as a response to cell death in the top level.

Self-renewal and differentiation events are assigned probabilistically, by means of the following process. Suppose *a*_*i*_ cells must be replaced in compartment *C*_*i*_, that is, there are *i* openings to fill. Each cell is replaced by a self-renewal division in this compartment with probability *v*_*i*_. If the number of remaining openings is odd, then an additional self-renewal division happens, such that the number of remaining openings is even, and they are then filled by differentiation divisions from compartment *C*_*i*−1_. This allows us to calculate the probability of a given tree defined by string (1). First we calculate the probability of having *a*_*i*_ differentiations in compartment *C*_*i*_ given that there are *a*_*i*+1_ differentiations in compartment *C*_*i*+1_:

$$\begin{array}{c} p\left(a_{i}\right)=C_{a_{i+1}}^{2a_{i}}\;{\left(1-v_{i+1}\right)}^{2a_{i}}v_{i+1}^{a_{i+1}-2a_{i}}+C_{a_{i+1}}^{2a_{i}+1}\;{\left(1-v_{i+1}\right)}^{2a_{i}+1}v_{i+1}^{a_{i+1}-2a_{i}-1},\;0\leq i\leq n-1, \end{array}$$

where formally *a*_*n*_ = 2^*n*^ and we use the following notation for the binomial coefficients:

$$\begin{array}{c} C_{n}^{k}=\frac{n!}{k!(n-k)!}. \end{array}$$

We then have the probability of string (1):

$$\begin{array}{c} P\left(a_{0},\ldots ,a_{n-1}\right)=\prod_{i=0}^{n-1}p\left(a_{i}\right). \end{array}$$

In particular, since *v*_*n*_ = 0, we obtain that *a*_*n*−1_ = 2^*n*−1^ with certainty.

**Fig 2:**
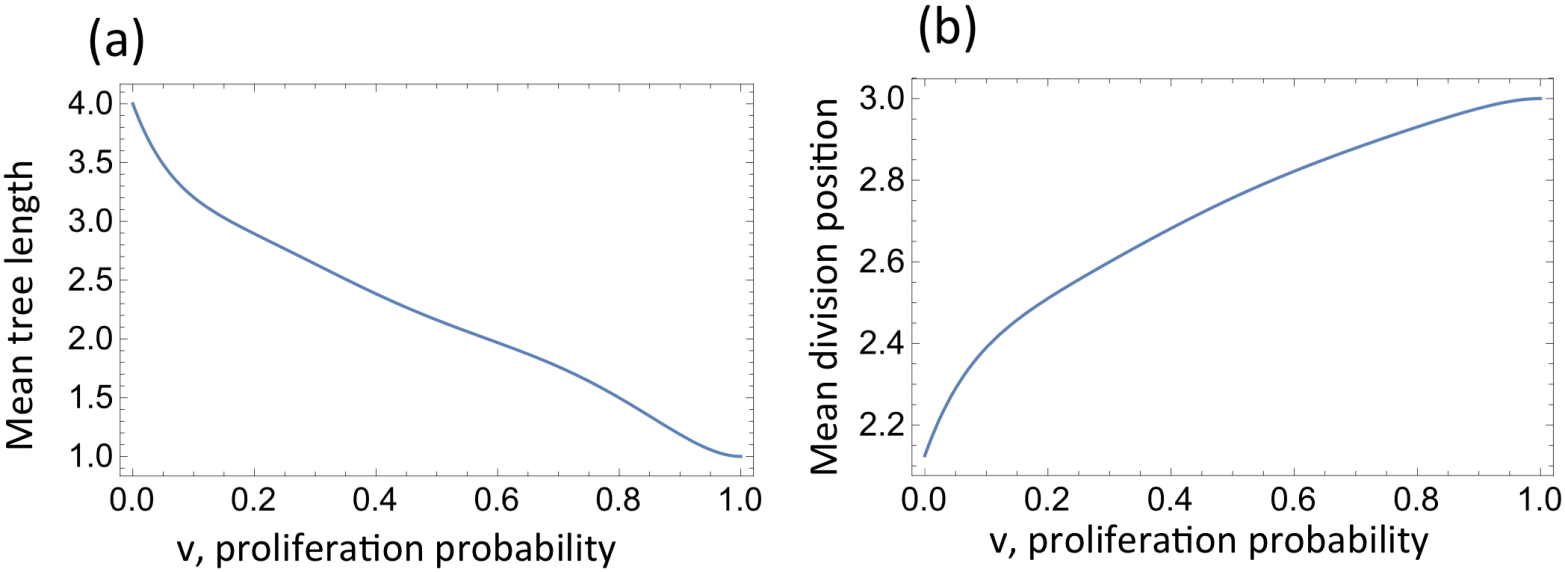
Properties of division trees as a function of *v*, the probability of self-renewal in compartments 1, …, *n* − 1. (a) Mean tree length, defined as the mean number of compartments involved in the division trees. (b) Mean division position. For an individual tree, sequence (1), this is defined as $2^{-n}\sum_{i=0}^{n}2i\left(a_{i}-a_{i-1}\right)$; plotted is the expectation of this quantity across all the trees. In this example, *n* = 4.

In Fig A in S1 Text and Fig B in S1 Text (Section 1 in S1 Text) examples are presented where probabilities of all possible division trees are calculated for a particular value of *n*. In general, decreasing the probability of self-renewal, *v*, increases the mean length of differentiation trees, meaning that divisions occur in the more primitive cell levels, see Fig 2.

### Deterministic dynamics of the expected number of mutants

We can formulate a metapopulation-style, ordinary differential equation (ODE) model of the renewal dynamics described above. Let us suppose that in each compartment, the number of mutants is given by *m*_*i*_, 0 ≤ *i* ≤ *n*. We would like to calculate the expected change in the number of mutants after 2^*n*^ cells are removed in compartment *C*_*n*_. To do this, let us determine the expected change in the number of mutants associated with a particular tree, string (1). This can be done by considering the change resulting from *a*_*k*_ differentiations from compartment *C*_*k*_ to compartment *C*_*k*+1_ and *a*_*k*+1_ − 2*a*_*k*_ self-renewals in compartment *C*_*k*+1_. These events will affect the numbers of mutants in compartments *C*_*k*_ and *C*_*k*+1_. Note that in the equations in this section we used combinatorial expressions obtained from sampling with replacement. In the stochastic simulations below, sampling without replacement was used, but it was established that sampling with replacement led to similar results.

(a) When *a*_*k*_ cells differentiate out of compartment *C*_*k*_, 0 ≤ *k* ≤ *n* − 1, with probability
  $$\begin{array}{c} P_{i}^{\text{diff}}=C_{a_{k}}^{i}\mu_{k}^{i}{\left(1-\mu_{k}\right)}^{a_{k}-i}, \end{array}$$
  *i* of them may be mutants, where
  $$\begin{array}{c} \mu_{k}=\frac{m_{k}}{N_{k}} \end{array}$$
  is the probability to pick a mutant among *N*_*k*_ cells. In this case, *i* mutants are removed from compartment *C*_*k*_ and 2*i* mutants are added to compartment *C*_*k*+1_. Further, among the remaining *a*_*k*_ − *i* differentiations, there may be *l* mutations which will increase the number of mutants in compartment *C*_*k*+1_ by *l*. To calculate the corresponding probability, we note that *a*_*k*_ − *i* wild type cells differentiating in compartment *C*_*k*_ will produce 2(*a*_*k*_ − *i*) offspring, each of which is a mutant with probability *u*. Therefore, *l* mutants are generated with probability
  $$\begin{array}{c} P_{i,l}^{\text{diff}}=C_{2(a_{k}-i)}^{l}u^{l}{\left(1-u\right)}^{2(a_{k}-i)-l}. \end{array}$$
  The total change in compartment *C*_*k*+1_ is then 2*i* + *l*, the total change in compartment *C*_*k*_ is −*i*, and this happens with probability $P_{i}^{\text{diff}}P_{i,l}^{\text{diff}}$. These changes must be summed up for 0 ≤ *i* ≤ *a*_*k*_ and 0 ≤ *l* ≤ 2(*a*_*k*_ − *i*).
(b) When *a*_*k*+1_ − 2*a*_*k*_ cells proliferate in compartment *C*_*k*+1_, 0 ≤ *k* ≤ *n* − 1, with probability
  $$\begin{array}{c} P_{i}^{\text{pro}}=C_{a_{k+1}-2a_{k}}^{i}\mu_{k+1}^{i}{\left(1-\mu_{k+1}\right)}^{a_{k+1}-2a_{k}-i}, \end{array}$$
  *i* of them are mutants. This means that *i* new mutants are added to compartment *C*_*k*+1_. The remaining *a*_*k*+1_ − 2*a*_*k*_ − *i* proliferating cells are wild type, and it is possible that *l* of the offspring are new mutants. This happens with probability
  $$\begin{array}{c} P_{i,l}^{\text{pro}}=C_{2(a_{k+1}-2a_{k}-i)}^{l}u^{l}{\left(1-u\right)}^{2(a_{k+1}-2a_{k}-i)-l}. \end{array}$$
  The total change in compartment *C*_*k*+1_ is then given by *i* + *l* and happens with probability $P_{i}^{\text{pro}}P_{i,l}^{\text{pro}}$. Again, these changes must be summed up for 0 ≤ *i* ≤ *a*_*k*_ and 0 ≤ *l* ≤ 2(*a*_*k*_ − *i*).
(c) A special case is compartment *C*_0_. Here, *a*_0_ cells will proliferate, and the number of mutants is calculated similar to (b).
(d) Another special case is compartment *C*_*n*_. Cells do not proliferate in this compartment, but 2^*n*^ cells are removed, and we need to calculate the expected number of mutants removed, similar to $P_{i}^{\text{diff}}$ in (a).

For each tree, the expected change in the number of mutants can be expressed as a vector whose components are obtained as the expected change in each compartment, as calculated in (a-d) above. These vectors must be added together, weighted by the probability of each tree. The resulting vector gives the time derivatives of the quantities *m*_0_, …, *m*_*n*_.

When *a*_*k*_ cells differentiate out of compartment *C*_*k*_, 0 ≤ *a*_*k*_ ≤ *n* − 1, the expected gain of mutants in compartment *C*_*k*+1_ is given by

$$\begin{array}{c} \alpha_{k+1}^{\text{diff}}=\sum_{i=0}^{a_{k}}\sum_{l=0}^{2(a_{k}-i)}\left(2i+l\right)P_{i}^{\text{diff}}P_{i,l}^{\text{diff}}. \end{array}$$

When *a*_*k*+1_ − 2*a*_*k*_ cells proliferate in compartment *C*_*k*+1_, 0 ≤ *k* ≤ *n* − 1, the expected gain of mutants in compartment *C*_*k*+1_ is

$$\begin{array}{c} \alpha_{k+1}^{\text{pro}}=\sum_{i=0}^{a_{k}}\sum_{l=0}^{2(a_{k}-i)}\left(i+l\right)P_{i}^{\text{pro}}P_{i,l}^{\text{pro}}. \end{array}$$

The compartment *C*_*k*+1_ loses mutants when *a*_*k*+1_ cells differentiate and migrate into compartment *C*_*k*+2_, and the expected loss is

$$\begin{array}{c} \beta_{k+1}^{\text{diff}}=\sum_{i=0}^{a_{k+1}}\sum_{l=0}^{2(a_{k+1}-i)}\left(2i+l\right)P_{i}^{\text{diff}}P_{i,l}^{\text{diff}}. \end{array}$$

Thus the total change in the number of mutants in compartment *C*_*k*+1_ is

$$\begin{array}{c} T_{k+1}=\alpha_{k+1}^{\text{diff}}+\alpha_{k+1}^{\text{pro}}-\beta_{k+1}^{\text{diff}}. \end{array}$$

The value *T*_*k*+1_ represents the expected number of mutants in compartment *C*_*k*+1_ produced by a single tree or chain of differentiation; because more than one tree could affect the expected number of mutants in each compartment, we need to sum the contributions from all trees to compartment *C*_*k*+1_ weighted by their probability. This weighted sum defines the rate of change of the number of mutants in each compartment:

$$\begin{array}{c} {\dot{m}}_{k}=\sum_{\text{All}\;\text{trees}}{\text{Prob}}^{\text{tree}}T_{k}^{\text{tree}},\;0\leq k\leq n. \end{array}$$ (2)

For the simplest cases, we list the ODEs derived by computing the expected number of mutants produced by all possible chains of differentiation in each compartment. We will use the assumption that *v*_*i*_ = *v* for 1 ≤ *i* ≤ *n* − 1, which has been assumed in other studies [7, 9, 23].

For the case *n* = 1 we have

$$\begin{array}{ccc} {\dot{m}}_{1} & = & u+\mu_{0}\left(1-u\right)-\mu_{1},\\ {\dot{m}}_{0} & = & 2u(1-\mu_{0}). \end{array}$$

For *n* = 2 we have

$$\begin{array}{ccc} {\dot{m}}_{2} & = & 4(u+\mu_{1}\left(1-u\right)-\mu_{2}),\\ {\dot{m}}_{1} & = & 2{\left(1-v\right)}^{2}\left(u+\mu_{0}\left(1-u\right)-\mu_{1}\right)+4u\left(2-v\right)v\left(1-\mu_{1}\right),\\ {\dot{m}}_{0} & = & 2u{\left(1-v\right)}^{2}\left(1-\mu_{0}\right). \end{array}$$

For *n* = 3 we have

$$\begin{array}{ccc} {\dot{m}}_{3} & = & 8(u+\mu_{2}\left(1-u\right)-\mu_{3}), \end{array}$$ (3)

m˙2=4[u(1-μ1+v(2-3v+4v2-2v3)(1+μ1-2μ2))=+(1-v)2(1+2v2)(μ1-μ2)], (4)

m˙1=2(1-v)6(u+μ0(1-u)-μ1)+4u(2-v)(1-v)4v(1-μ1)=+4uv(2-3v+v3)(1-μ1), (5)

$$\begin{array}{ccc} {\dot{m}}_{0} & = & 2u{\left(1-v\right)}^{6}\left(1-\mu_{0}\right). \end{array}$$ (6)

To create a system for a general value of *n*, and also for general, non constant values of *v*_*i*_, we have created a program in *Mathematica*, see S1 File. In Section 1.2 in S1 Text, we present further details of these calculations.

The *n* + 1 linear ordinary differential equations derived here are applicable in the high mutation rate / large population limit (*uN* ≫ 1). The unique fixed point of the system, for all *n*, which is always stable, is

$$\begin{array}{c} m_{i}=N_{i},\;0\leq i\leq n. \end{array}$$

**Fig 3:**
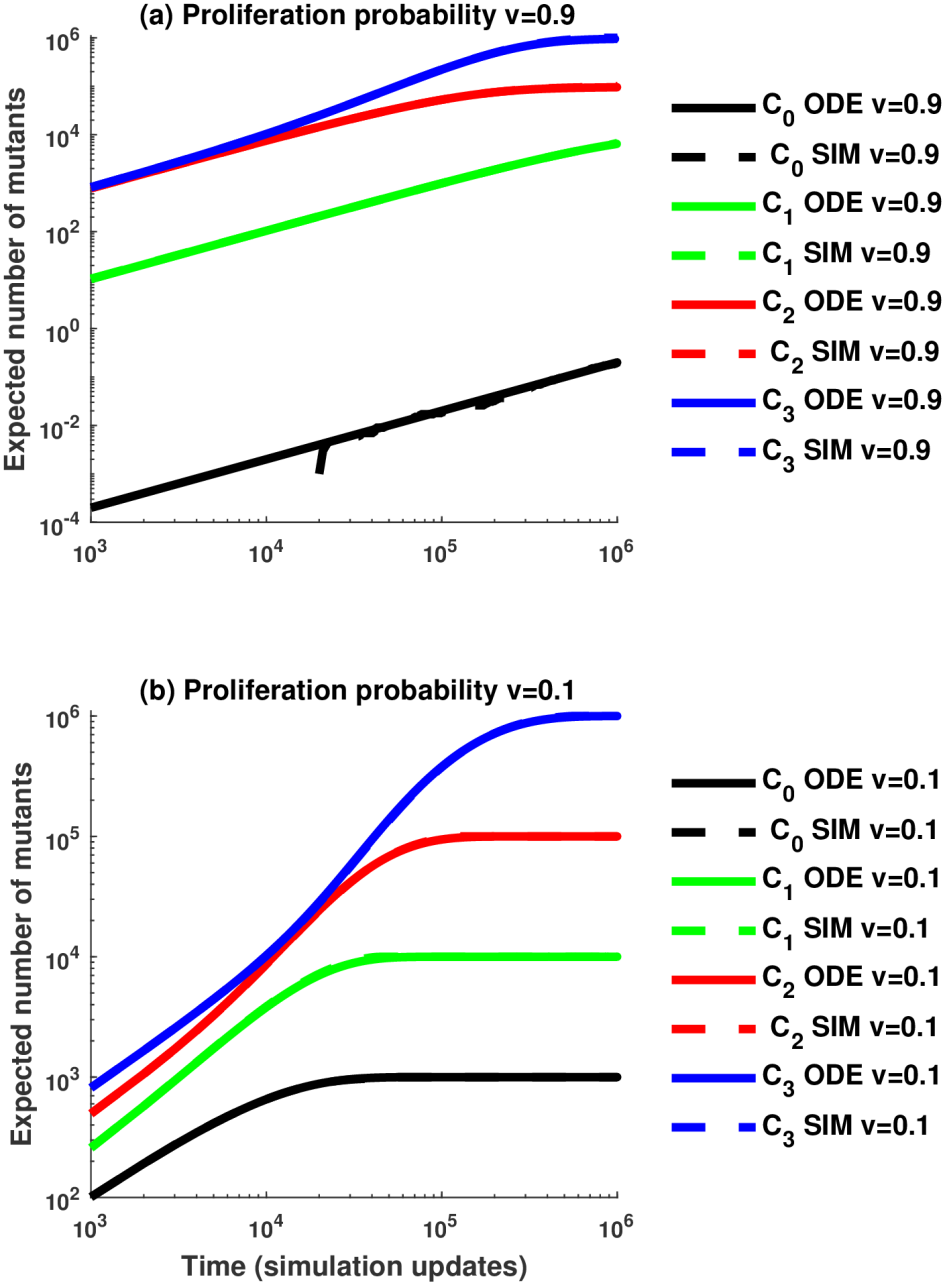
Comparison between the average number of mutants from 100 stochastic simulations without replacement and the number of mutants predicted by the ODE approximation. (a) Comparing the number of mutants for a high value of the proliferation probability, *v* = 0.9. (b) Comparing the number of mutants when the proliferation probability is small, *v* = 0.1. Solid lines correspond to the number of mutants in compartment *C*_*i*_, *i* = 1, …, 3, predicted by the ODE approximation and dashed lines correspond to the mean number of mutants in compartment *C*_*i*_ from the stochastic simulations. We assume *n* = 3, a mutation rate of *u* = 10^−3^, and the compartment sizes are *N*_0_ = 1063, *N*_1_ = 10^4^, *N*_2_ = 10^5^, *N*_3_ = 10^6^.

In Fig 3, we set *n* = 3 and show the number of mutants in each of 4 compartments as a function of time, obtained from numerical solution of deterministic system (3–6) (solid lines) and stochastic simulations (dashed lines). The dynamics for two different values of *v* are presented, *v* = 0.9 and *v* = 0.1; the numerical and simulated results in both scenarios are very close and for the most part are impossible to distinguish. The number of mutants reaches saturation, *N*_*i*_, in each compartment. The larger the value of *v*, the longer it takes for the lower compartments to reach their equilibrium values. This is because for large self-renewal probabilities, long trees are unlikely and divisions rarely happen in the less differentiated compartments. Further details of the deterministic regime are presented in S1 Text. Note that in this paper, we use ODE’s as a deterministic tool to study the stochastic system at hand. An alternative approach was adopted by [23] who used ODEs as the basic model and studied the stochastic effects by formulating a related Gillespie process.

## Results

The central question we address is what values of the self-renewal probability, *v*, produce fewer mutants; related to this, we investigate how the length of differentiation chains affects the accumulation of cancer cells. If low values of *v* produce fewer mutants, this would imply that long chains of differentiation are favorable, as they are likely to decrease the number of cancer cells; on the other hand, if high values of *v* produce fewer mutants, short chains of differentiation would decrease the number of cancer cells. In addition, we investigate how tissue architecture combined with the self-renewal probabilities affects mutant production.

### De-novo mutant generation

In the ODE model derived here, mutations are generated constantly at a rate *u*. This is a correct assumption with high mutation rates and high population numbers. In smaller systems with low mutation rates, the processes of mutant (de novo) generation and their clonal spread can be treated as separate. Mutant generation becomes a rare event, and a single mutant that is generated gives rise to a clone that does not interfere with the creation of other mutant clones. This regime can also be studied with the help of the probabilities calculated above.

The removal of 2^*n*^ mature cells from compartment *C*_*n*_ gives rise to a chain of 2^*n*^ cell divisions that replenishes the lost cells. The probability of each such chain is calculated above. Further, every chain gives rise to an expected change in the mutant numbers in each of the compartments. This information can be used to study the de-novo generation of mutants and their subsequent clonal spread. To calculate the probability that a mutant will be generated in each of *n* + 1 compartments, we evaluate the right hand side of system Eq (2) with *m*_*i*_ = 0 for all 0 ≤ *i* ≤ *n* (that is, no prior mutations exist in the system). We obtain a vector proportional to the mutation rate *u*. Normalizing this vector to create a probability distribution, we obtain the probability to generate a mutant in each of the compartments. This vector only depends on the quantities (*v*_1_, …, *v*_*n*−1_), and not on the population sizes of individual compartments.

**Fig 4:**
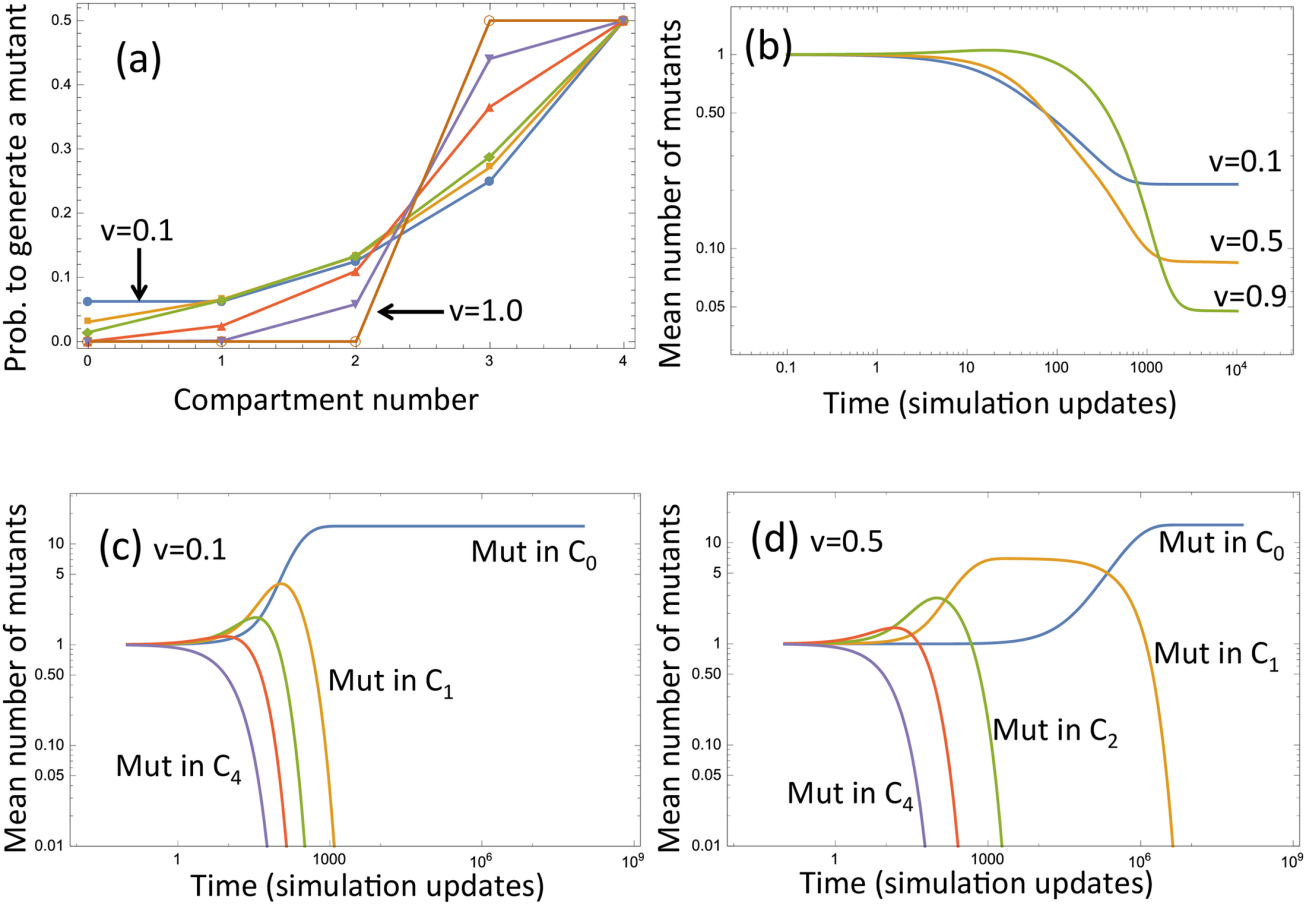
The role of the self-renewal rate on mutant generation and mutant dynamics (the analytical approach). (a) The probability of generating a mutant in each of the compartments for 6 different values of *v*: *v* = 0, 0.05, 0.1, 0.4, 0.7, 1. (b) The expected number of mutants produced from a single mutant cell in the absence of further de-novo mutations, plotted as a function of time for three different values of *v*. (c,d) The expected dynamics of mutants generated in different compartments at *t* = 0, in the absence of new mutations, for (c) *v* = 0.1 and (d) *v* = 0.5. Other parameters are: *n* = 4, and the compartment sizes are, from *C*_0_ to *C*_*n*_: 40, 80, 120, 160, 200.

We can study the vector of probabilities of mutant generation by setting *v*_*i*_ = *v* for all 1 ≤ *i* ≤ *n* − 1 and examining the dependence on *v*. Smaller values of *v* give rise to longer trees, and consequently higher probabilities of generating mutants in the stem cell compartment. Larger values of *v* correspond to shorter trees, such that mutants are not generated in the stem cell compartment. These trends are illustrated in Fig 4(a).

### Clonal dynamics of mutants

Once generated, a mutant undergoes clonal expansion and is also subject to being flushed out of the system (depending on its location). Let us consider the clonal propagation of mutants, in the absence of new mutations, which is achieved by setting *u* = 0 in the main system of ODEs, Eq (2). We obtain the following system:

$$\begin{array}{c} {\dot{m}}_{0}=0,\;{\dot{m}}_{k}=K_{k}^{\left(v\right)}(\frac{m_{k-1}}{N_{k-1}}-\frac{m_{k}}{N_{k}}),\;k\geq 1, \end{array}$$ (7)

where the constants $K_{k}^{\left(v\right)}$, which we can call the “flush-out rates”, depend on the vector (*v*_0_, …, *v*_*n*_). One can show that these rates scale with powers of (1 − *v*). For example, for compartment *C*_1_ and for constant *v*, the flush-out rate is given by

$$\begin{array}{c} K_{1}^{\left(v\right)}=2{\left(1-v\right)}^{2^{n}-2}, \end{array}$$ (8)

see Section 1 in S1 Text. Generally, for constant *v*, we have the following expansion of the flush-out rates in the vicinity of *v* = 1:

$$\begin{array}{c} K_{i}^{\left(v\right)}=A_{i}{\left(1-v\right)}^{\gamma_{i}}+O{\left(1-v\right)}^{\gamma_{i}+1},\;1\leq i\leq n, \end{array}$$

where *γ*_*i*_ = 2^*n*−*i*+1^ − 2 and *A*_*i*_ does not depend on *v*.

The structure of Eq (7) can be understood intuitively. Suppose that *Q* cells are removed from compartment *C*_*k*_. This results in three effects:

- The expected number of mutants removed from *C*_*k*_ by differentiating out is *Qμ*_*k*_.
- On average, *Qv*_*k*_ cells will be replaced by self-renewals in compartment *C*_*k*_, resulting in *Qv*_*k*_*μ*_*k*_ mutants added on average to *C*_*k*_.
- The remainder of the cells, *Q*(1 − *v*_*k*_), will be replaced by differentiations from compartment *C*_*k*−1_. There will be *Q*(1 − *v*_*k*_)/2 such differentiations, resulting in *Q*(1 − *v*_*k*_)*μ*_*k*−1_ mutants added on average to compartment *C*_*k*_.

The net change in the mean number of mutants is then given by *Q*(1 − *v*_*k*_)(−*μ*_*k*_ + *μ*_*k*−1_), which agrees with the above equations. The steady state of system Eq (7) is given by

$$\begin{array}{c} m_{k}=m_{0}\frac{N_{k}}{N_{0}}. \end{array}$$ (9)

The interpretation of this result is quite straightforward: the probability of fixation of the mutant in compartment *C*_0_ is *m*_0_/*N*_0_, and the expected number of mutants in all the downstream compartments is given by the corresponding compartment size multiplied by this fixation probability.

Depending on the origin of the mutation, the behavior of this system (and therefore, mutant clonal dynamics) will be different. Only in the case where the mutant is introduced at *C*_0_, is the steady state Eq (9) nontrivial, that is, mutations will persist in the system if the stem cell compartment acquires a mutation. If a mutant is introduced in any other compartment, there is transient dynamics, and then the mutation will be flushed out, since *m*_0_ = 0 in Eq (9).

The diagonal entries of the lower-triangular matrix in the linear system Eq (7) define the time-scale of the transient dynamics. The first diagonal entry is zero and corresponds to the neutrality of the number of mutants in the stem cell compartment. This is a consequence of modeling the behavior of mutants deterministically: in a stochastic system, the probability of increasing and decreasing the number of mutants in *C*_0_ are equal to each other, leading to a symmetric Markov chain. The mean behavior however is correctly captured by the present system. The rest of the diagonal entries of the matrix in system Eq (7) have quantities *N*_*i*_ in the denominators (that is, larger compartments are characterized by slower mutant dynamics), and the numerators are products of powers of 1 − *v*_*k*_. For small values of the self-renewal probabilities, the mutants are flushed out quickly because of a high rate of differentiations leading to upward motion of cells through the compartments. For larger values of *v*, mutants are flushed out slowly so they tend to accumulate in compartments, giving rise to very long-lived mutant populated states. In the extreme case of *v* = 1, only compartment *C*_*n*−1_ divides, and mutants accumulate in that compartment (the right hand side of Eq (7) is zero in this case, except for the equation for *m*_*n*_). These trends are illustrated in Fig 4(c) and 4(d), where the dynamics of mutant clones are shown depending on the compartment of mutant origin. These results are obtained from the ODEs in Eq (7).

### The role of self-renewal in mutant generation and dynamics

As the value of *v* increases, there is a trade-off between two different trends associated with the probability of self-renewal: the shortening of the division trees on the one hand, and a decrease in the mutant flush-out rate. More precisely, small values of *v* imply longer trees and a higher probability of generating mutants in the stem cell compartment. When the mutants arise however, they will be flushed out quickly. On the contrary, large values of *v* correspond to shorter trees, such that mutants are not likely to be generated in the stem cell compartment. Once generated, however, the mutants accumulate and persist for longer. These trends are illustrated in Fig 4(b). There, we plot the expected dynamics of mutants, where the initial condition reflects the probabilities of producing a mutant in the different compartments, Fig 4(a). We assume that the initial condition is a probability vector from Fig 4(a), no further mutants are generated, and clonal dynamics are according to Eq (7). We observe that for small values of *v*, mutant accumulation is initially the least, but for larger times, there is a significant expected number of mutants in steady state (that is, a good chance that the mutants fixate). For larger values of *v*, mutants tend to accumulate (as shown by the transient increase in the number of mutants), but in the long run, the expected number of mutants is smaller (a smaller chance that there is fixation in the system). While the above examples assume a constant value of *v*, a more general result holds (see Section 1.2 in S1 Text): the flush-out rates $K_{i}^{\left(v\right)}$ are non-increasing functions of all self-renewal probabilities *v*_*i*_ (see Fig B in S1 Text), and the probability of mutant creation in compartment *C*_0_ is a decreasing function of the values *v*_*i*_.

In S1 Text we also explore how compartment sizes influence mutant generation and expansion (Section 7 in S1 Text), the effect of asymmetric divisions (Section 5 in S1 Text) and the developmental stage (Section 4 in S1 Text).

### Stochastic simulations and a summary of trends

**Fig 5:**
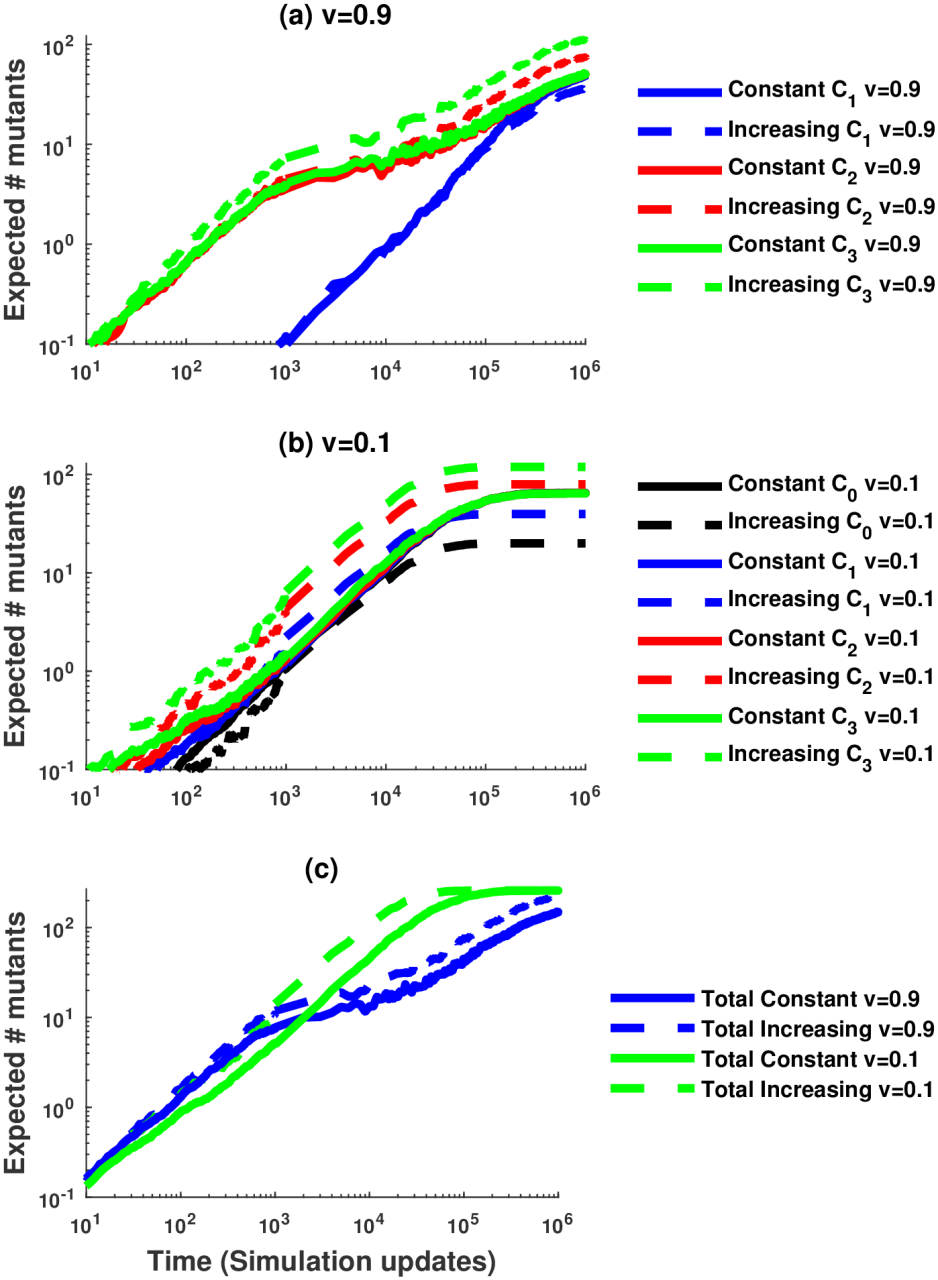
Mean number of mutants from 1000 stochastic simulations. We compare two arrangements of the compartment sizes for *n* = 3: constant from *C*_0_ to *C*_3_ (*N*_0_ = 65, *N*_1_ = 65, *N*_2_ = 65, *N*_3_ = 65) and increasing from *C*_0_ to *C*_3_ (*N*_0_ = 20, *N*_1_ = 40, *N*_2_ = 80, *N*_3_ = 120). (a) The mean number of mutants produced in compartments *C*_1_, *C*_2_ and *C*_3_ for *v* = 0.9. Note that no mutants were produced in compartment *C*_0_ over the time scale shown. (b) The mean number of mutants produced in compartments *C*_0_, *C*_1_, *C*_2_ and *C*_3_ for *v* = 0.1. (c) The mean of the total number of mutants comparing both architectures for *v* = 0.9 (blue line) and *v* = 0.1 (green line). For all panels, solid lines correspond to constant architecture and dashed lines to increasing architecture. In these simulations *u* = 0.001.

In this study, the number of mutants produced by a stem cell lineage system is influenced by different factors. The two factors that we focused our investigation on are the probability of self-renewal in the compartments, *v*, and the size of different compartments. Recall that low probability of self-renewal translates into activation of many upstream cell compartments, which is equivalent to having longer division trees, while high probability of self-renewal translates into activation of few upstream cell compartments, which is equivalent to having shorter division trees. Here we summarize our findings and further illustrate them with stochastic simulations, Fig 5. In this figure, we present the mean dynamics of mutations in different compartments, corresponding to two different values of *v* and two different architectures, increasing and constant. As shown below, the behavior of the stochastic system is consistent with our predictions based on the ODEs, which were described above.

1. **Self-renewal probability and accumulation of mutants.** In the short term, lower probabilities of cell self-renewal (lower values of *v*) tend to be advantageous, as they result in a lower overall number of mutants; for larger values of *v* mutants tend to accumulate quickly, which temporarily increases the number of mutants beyond what is observed in low-*v* systems, see Figs 5(c) and 4(b). As *v* increases and the mean tree length decreases, there is a trade-off between decreasing the probability of mutant creation in the SC compartment and also decreasing the flush-out rate. In the long run, however, we observe that the latter tendency becomes less important, and larger values of *v* result in a smaller overall number of mutants. This can be seen again in Fig 4(b). The same tendency is observed in stochastic simulations of Fig 5(c), where blue lines (*v* = 0.9) are below green lines (*v* = 0.1) for large times. The typical time-scale before the equilibrium is reached scales as 2^−*γ*^ with *γ* = 2^*n*^ − 2.
2. **Self-renewal probability and mutations in stem cell compartment.** Larger values of *v* are again advantageous from the viewpoint of minimizing the number of mutants in the stem cell compartment. With a shorter division tree length, divisions in the stem cell compartment happen less often leading to a lower number of mutations. This can be seen for example in Fig 3 by comparing the black lines (the number of mutants in compartment *C*_0_) and in Fig 4(a); see also Fig 5, where for the time scale of the simulations, no mutants have appeared in *C*_0_ in (a) for *v* = 0.9, and there is a nonzero expected number of mutants in *C*_0_ in (b) for *v* = 0.1 (depicted by black lines).
3. **Compartment sizes and accumulation of mutants.** When the value of *v* is fixed, constant architectures (where all compartment sizes are equal, marked “constant” in the figures) will ultimately produce fewer mutants than increasing architectures; see Fig 5(c) here in and Fig J(b) in S1 Text. However, as shown in Fig J(a) in S1 Text, increasing architectures (when compartment size increases in the downstream direction) will appear to perform better than constant architectures for a short initial period with large *v* values. Thus, while constant architectures are more beneficial in the long-term, increasing architectures are beneficial in the short-term when *v* is large. Another scenario where increasing compartment sizes are advantageous arises if we assume that the self-renewal probabilities in compartments are correlated with compartment sizes, such that differentiation from large to small compartments is favored. In this case, increasing architecture may give rise to fewer mutants, see Fig J(b) in S1 Text.
4. **Compartment sizes and mutations in stem cell compartment.** Increasing architecture always minimizes the number of mutants in the stem cell compartment. This follows from the ODE description, see for example, the equation for the number of mutants in compartment *C*_0_, Eq (6). The same trend is observed in the stochastic dynamics, see the black lines in Fig 5(b), where the dashed line corresponding to the increasing architecture shows fewer mutants in *C*_0_ compared to the solid line for constant architecture (please note that in this figure, in the long run, the number of mutants in each compartment in the constant architecture is very similar, such that all the solid lines are superimposed).

Finally we note that a model that includes asymmetric divisions is presented in Section 5 in S1 Text.

### Generation of two-hit mutants

**Fig 6:**
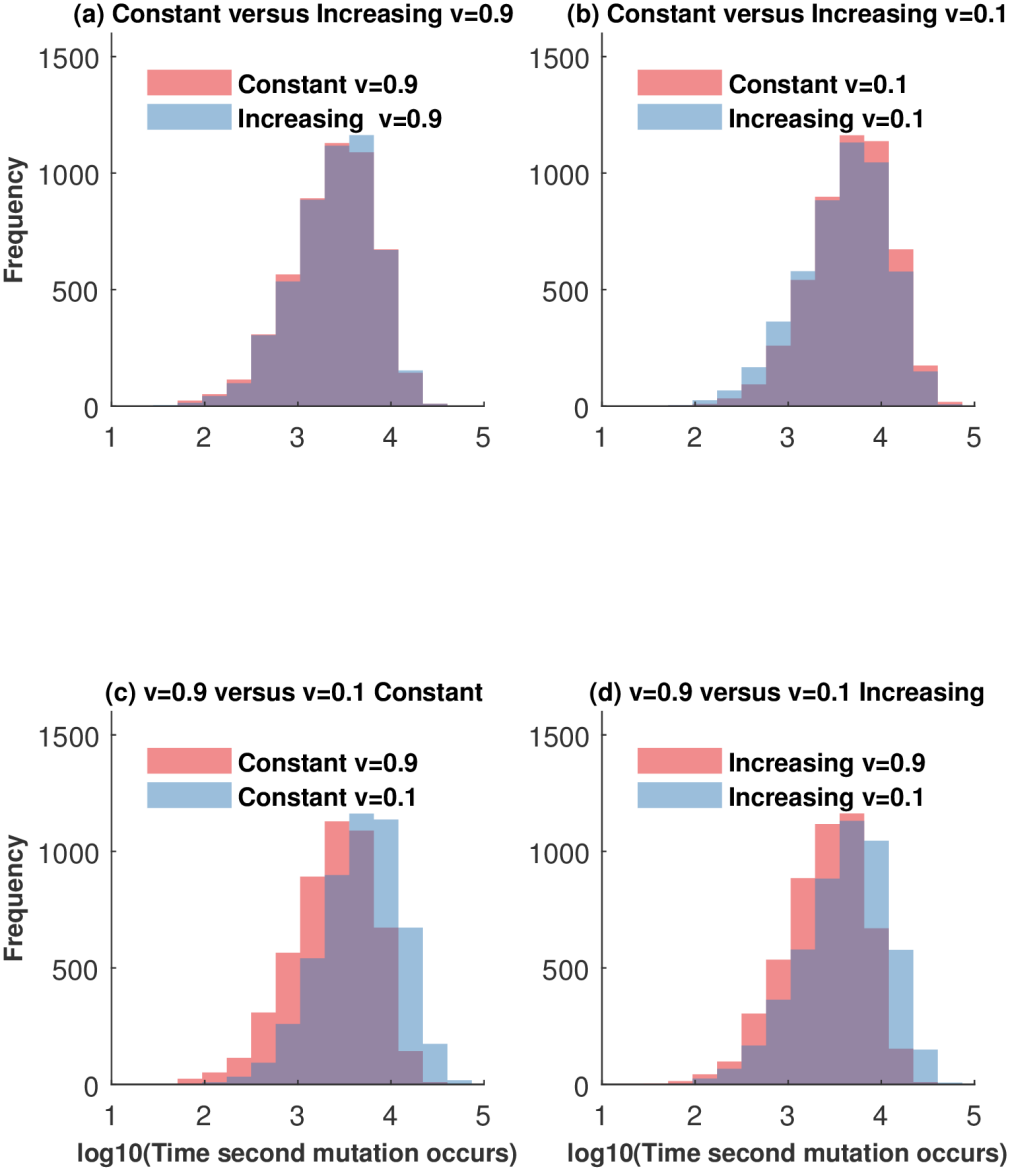
Distribution of the generation times to second mutation from 5000 stochastic simulations. We consider two arrangements of the compartment sizes for *n* = 3: constant from *C*_0_ to *C*_3_ (*N*_0_ = 65, *N*_1_ = 65, *N*_2_ = 65, *N*_3_ = 65) and increasing from *C*_0_ to *C*_3_ (*N*_0_ = 20, *N*_1_ = 40, *N*_2_ = 80, *N*_3_ = 120). (a) The time to observe a second mutant for both architectures and a high value of the self-renewal probability, *v* = 0.9. The mean time for constant and increasing architectures is 3.33 and 3.39 respectively; the *p*-value obtained by two-tailed *t*-test is *p* = 0.078, indicating that the means are different only at the 10% level (size effect is 0.04). (b) The time to observe a second mutant for both architectures and a small value of the self-renewal probability, *v* = 0.1. The mean time for constant and increasing architectures is 3.67 and 3.60 respectively; the *p*-value is *p* ≪ 0.001, indicating that the means are different; the effect size is 0.16. (c) and (d) The time to observe a second mutant for a constant and increasing architecture, respectively, for small and high *v*. In these simulations *u* = 0.001, and the effect size is 0.68 and 0.46 respectively.

Stochastic simulations developed here were also used to investigate the dynamics of two-hit mutant generation. In this setting, one-hit mutants were allowed to undergo a secondary mutation process, and the simulations were stopped as soon as the first two-hit mutant was generated. This situation corresponds to the scenario where two-hit mutants are advantageous and do not obey the same homeostatic control as the wild type cells or one-hit mutants. Fig 6 shows the histograms of times when the first two-hit mutant was generated under different parameters. It presents a comparison between different tissue architectures (panels (a) and (b)) and different self-renewal probabilities (panels (c) and (d)) s. In particular, we notice that the largest difference in two-hit mutant generation times is experienced when we change *v*; see Fig 6(c) and 6(d). For both architecture types, low values of *v* lead to longer two-hit mutant generation times. Therefore, we can say that from the viewpoint of two-hit mutant generation, low values of *v* are advantageous. This can again be explained by thinking about flush-out rates. Larger values of *v* lead to long-lived (although transient) mutant accumulation in tissue compartments, which in turn results in a higher chance of two-hit mutant generation. As a consequence, having lower self-renewal rates and longer differentiation trees results in slower two-hit mutant generation.

It has been observed that small differences in fitness can be acted upon by selection [34, 35]. The differences in tissue architectures result in smaller differences in two-hit mutant generation time, but the trends we observe coincide with those seen in Fig J in S1 Text: for small values of *v*, increasing architecture leads to a faster production of two-hit mutants (because it results in the fastest accumulation of one-hit mutants), and for larger values of *v*, increasing architecture corresponds to the largest delay of two-hit mutant generation. This effect becomes even stronger if asymmetric divisions are included in the model, see Fig I in S1 Text.

**Fig 7:**
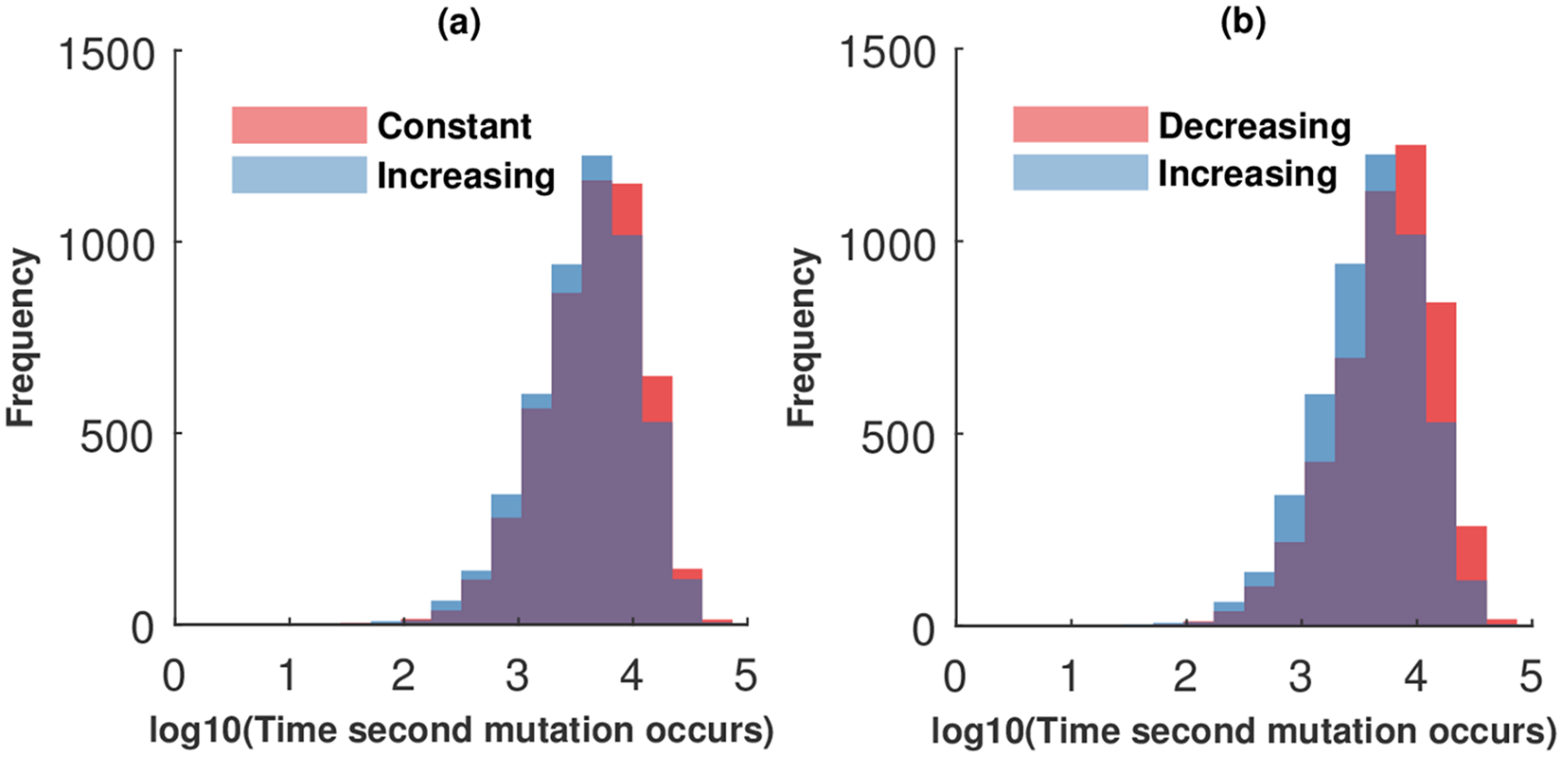
Distribution of the generation times to second mutation, obtained from stochastic simulations, in the case where *v* depends on the compartment sizes. We perform 5000 stochastic simulations and compare two arrangements of the compartment sizes: (a) constant compartment size (red bars) and increasing (blue bars); (b) decreasing (red bars) and increasing (blue bars). Compartment sizes are as in Fig 6. The self-renewal probabilities are *v*_1_ = 1, *v*_3_ = 0, $v_{i}=\frac{N_{i}}{N_{i}+N_{i-1}}$, *i* = 1, 2. We assume *n* = 3 and *u* = 0.001. In part (a), the mean time to a two-hit mutant for constant and increasing architecture is 3.65 and 3.59 respectively; the *p* value obtained by a two-tailed *t*-test is *p* ≪ 0.001, the effect size is 0.12. In part (b), the mean time to a two-hit mutant for decreasing architecture is 3.73; *p* ≪ 0.001; the effect size is 0.30.

Fig 7 further explores the differences between increasing and constant architecture by assuming that the self-renewal probability is defined by the compartment sizes. We observe that the constant architecture tends to delay the production of two-hit mutants compared to an increasing architecture. This result follows from the fact that lower values of *v* slow down two-hit mutant generation, as demonstrated above. Since the constant architecture results in lower values of *v*, it results in the longest two-hit mutant generation times.

## Discussion

In this paper we considered the dynamics of mutant accumulation in hierarchical tissues under homeostatic turnover. What tissue architecture can be considered optimal from the viewpoint of minimizing mutations? We have focused our attention on two aspects of tissue architecture: (1) probability of self-renewals/differentiations of cells in compartments; and (2) compartment size.

The first aspect is the probability of cells in each compartment to proliferate. A self-renewal division is one way of replenishing cells that have been removed (by differentiation) from the compartment; the other way is to replace “missing” cells by a differentiation division from an upstream compartment. Therefore, by changing the probability of self-renewal *v* (as opposed to differentiation) we change the probability that an upstream compartment will be engaged, thus changing the length of a typical hierarchical division tree. We have found that increasing *v* results in two clear tends: (i) the trees get shorter and it becomes unlikely that mutations are acquired in the more primitive compartments, and (ii) mutations that are generated are flushed out more slowly from the non-stem cell compartments, and tend to accumulate (at least, transiently). This interesting trade-off results in different “optimal” solutions, depending on the objective of optimization. If the goal is to minimize the total number of one-hit mutants residing long-term in all compartments, relatively large values of *v* are desirable. If on the other hand we want to protect the stem cell compartment, then low values of *v* are superior. Finally, from the point of view of two-hit mutant generation, again lower values of *v* are advantageous.

Although the optimization problem solved in this paper is formulated differently from the work presented by Dérenyi et al. in [27], there is an interesting point of convergence between our results and the results of [27]. The optimal architecture for delaying 2nd mutations corresponds to low values of *v*. Therefore, it is characterized by the longest possible active trees. These trees look like binary trees, which are (nearly) the perfect solution found by [27].

Maximally delaying the generation of two-hit mutants is important in the context of tumor suppressor gene inactivation, which is a common event in carcinogenesis. In tumor suppressor genes, such as the APC gene that is inactivated in a large fraction of colorectal cancers, or Rb gene responsible for retinoblastoma, both copies must be inactivated for the resulting cell to acquire a phenotypic change that eventually may lead to cancer. Tumor suppressor gene inactivation is often considered an early event in cancer progression, and therefore a delay in this event is of crucial importance for evolution. Our analysis indicates that in order to delay tumor suppression gene inactivation in a hierarchical tissue, mitotic activities must be concentrated near the stem cell compartment. This finding coincides with *in vivo* measurements performed in [36, 37], where divisions were more often observed near the bottom of the hierarchical compartments. In Section 7 in S1 Text, we compare the cell division data presented in [37] to the expected number of divisions generated by our model. The model output is only consistent with the data under the assumption of relatively low values of the self-renewal probability *v*, supporting our prediction that low values of *v* should be selected for.

Other, indirect pieces of evidence pointing toward the protective potential of cell division regulation come from studies that investigate the connection between loss of differentiation and cancer. Our result that small values of the self-renewal probability *v* lead to double-hit mutant suppression is consistent with the findings of [38], who reported that overexpression of protein kinase *Cβ*_*II*_ induced colonic hyperproliferation, and thus, increased the risk of colon carcinogenesis. This behavior has also been observed in other types of cancers, where a disruption in differentiation patterns might increase the risk of cancer [33, 39–42]. Tenen [43] explained that the loss of differentiation is an important component of many cancers, specifically, haematopoietic transcription factors are crucial for differentiation to particular lineages, and their disruption is critical in acute myeloid leukemia (AML) development. Researchers have also found that mutations related to brain cancer and leukemia cause the production of an enzyme that can reconfigure on–off switches across the genome and stop cells from differentiating [44–46].

To test model predictions about the accumulation of mutations more directly, we propose measuring the natural flush-out rate of cells in a hierarchical tissue, such as the colonic crypt. One approach to this is to use an experimental animal model and label a single cell (in the stem or other compartment). The cell’s progeny should then be tracked in the hierarchy through time using prospective lineage tracing. While often this is done at a single time point, observations at multiple time points would allow for a comparison with the clonal expansion of mutant (“labeled”) cells predicted by the model. While experimentally intensive, there are a variety of genetic techniques for prospective cell lineage tracing in model organisms that could be used to generate such data (see [29] for an example in the mouse intestinal crypt and [47] for a recent review).

We should also discuss this result in the context of the previous theoretical work by [7] and [48]. In both of these papers, variants of a linear model of the colon were investigated, where individual cells were arranged in a linear array and where dividing cells pushed out their neighbors upstream [49]. Interestingly, while both papers found that divisions near the stem cell compartment (the “bottom-high” division distribution) resulted in lowering the risk of secondary mutations experienced by existing one-hit mutants, the overall two-hit mutant generation was found by [48] to be accelerated in this case (and the “top-high” division distribution, where most divisions happen near the top, was shown to lead to a delay in two-hit mutant production). This pattern is different from the result reported in the present model, and the difference stems from the modeling assumptions: the linear model was adopted by [7, 48], whereas a model with separate compartments that experience a certain degree of mixing was considered here (similar in spirit to metapopulation models that are often used in population biology, see e.g. [50–53]). In the present model, the linear arrangement is not 1-cell thick, but instead, there is a possibility that mutants can accumulate, or “get stuck”, in a compartment that has a size greater than one cell. This is in contrast to the linear model, where such a possibility does not exist, and each mutant will inevitably be pushed toward death at the top of the crypt in a regular fashion. The tendency of mutants to accumulate in the presence of a large self-renewal probability discovered in the present setting, points toward a possibility that is ignored in the linear models. In a sense, this is similar to the differences between one-dimensional spatial population models on the one hand, and two-/three-dimensional population models or metapopulation models on the other, see also [54, 55] for discussion of these issues. Mutant spread is greatly restricted in one-dimensional geometry, whereas there are different ways to spread in higher dimensions or in metapopulations.

The second aspect of architecture investigated here is the arrangement of compartment sizes. It is physiologically determined that the top compartment, containing the most mature cells performing their function in the organ, must be the largest. Under this restriction, there are a great variety of ways in which sizes of consecutive compartments can be arranged. In this paper we show that the size of the upstream compartments matter when it comes to delaying mutant production in a hierarchical tissue. We found that the smaller the differences in the relative compartment size, the longer it takes on average to generate a double-hit mutant. This suggests the existence of evolutionary pressure to equalize compartment sizes to facilitate the flushing out of mutant cells.

Next, we would like to interpret our model and its implications in the broader context of tissue architecture and the development of multicellular organisms. We can view tissue as the “functional unit” consisting of the physiologically necessary, fully mature cells, and a “support unit” consisting of less differentiated cells that do not perform the same function but are there to support and replenish the cells from the functional unit. The question then becomes, how to best organize the support unit that maintains a certain number of “functional” cells, see also [5, 56]. As one extreme scenario, one can envisage a great number of very short division trees where more or less each mature cell is replenished by divisions of its own stem cell. At the other extreme, it could happen that all the mature cells are replenished by means of an enormously long division tree, and are all part of the same large lineage stemming from a common stem cell. Our model is capable of distinguishing between these two extreme possibilities (and the whole range of intermediate possibilities). If 2^*n*^ cells are removed, the longest division tree has length *n* and the shortest has length 1. If *v* = 0, then the longest tree is always utilized. If *v* = 1, then only the shortest trees are activated, and only cells in one upstream compartment (compartment *C*_*n*−1_) divide. If this was the evolutionarily preferable scenario, this would mean that compartment *C*_*n*−1_ is essentially the SC compartment, and that only one division step separates SCs from fully differentiated cells. If an intermediate value of *v* was selected, then the size of stem cell lineage would be defined by the length of a typical division tree corresponding to this value of *v*, and the upstream compartments that hardly ever divide would eventually be eliminated. Our results point towards the evolutionary utility of lengthening of stem cell lineages, because decreasing *v* leads to an increase in the two-hit mutant generation time. This process of lengthening of stem cell lineages however has to be limited by other factors, such as geometric constraints and the necessity of spatial distribution of lineages (for example colon crypts).

Incidentally, our result whereby lengthening of the SC lineages is preferred, is again different from the previous one obtained in [56], for the same reason that was highlighted above: the present model allows for mixing in the compartments, whereas the older model of [56] is rigid in the same way as the linear model of [7] (although it is not a linear model but rather a sequence of deterministic divisions on a binary tree, where randomness only comes about when creating de-novo mutations, see [17]).

The hierarchical model considered here, although less rigid in terms of division patterns compared with the linear model of [7] and the binary model of [56], is obviously still a simplification of reality. There are many extensions that can be introduced into this model. Somatic mutations are not necessarily neutral, they might be disadvantageous, or advantageous. In this context, a fitness advantage or disadvantage might be assigned to mutants to study how it impacts the mutant population. Another important feature that could be added to our model is a replication capacity. This could be done by assigning a maximum number of divisions to each compartment, which would definitely have an impact on the type of chains of differentiation observed and therefore, on mutant development. It has been shown that a finite, and relatively small, replication capacity (between 50 and 70 divisions [57]) is a mechanism of protection against cancer in hierarchically organized tissues [32].

Finally, we note that the present model takes the metapopulation approach to spatial relationships among cells. It can be refined by adopting a fully spatial, two- or three-dimensional description, for example, a cellular automaton or a hybrid model with a degree of cell migration included. It will be interesting to see how the properties of this model change under such a refinement. The main finding of the present model is that lowering the probability of self-renewal in each compartment and compensating by differentiations in longer division trees leads to a delay in two-hit mutant generation. We predict that this result should continue to hold in the fully spatial case.

## Supporting information

S1 Text A document containing additional calculations, numerical simulations, and figures, that further illustrate points made in the main text. (PDF)

S1 File A *Mathematica* file that allows to generate, for a given value of *N*, a list of all division trees, to calculate their probabilities, and evaluate the expected change in the number of mutants resulting from cell divisions that proceed according to each tree. (NB)
