# Supplementary material for: Optimizing homeostatic cell renewal in hierarchical tissues
Source: PLoS Comput Biol. 2018 Feb 15;14(2):e1005967. doi: 10.1371/journal.pcbi.1005967 (PMC5831642; doi:10.1371/journal.pcbi.1005967)
Supplement: S1 Text — (PDF) [file pcbi.1005967.s001.pdf]

S1 Text

Optimizing homeostatic cell renewal in hierarchical tissues

Cesar Alvarado, Nicole A. Fider, Helen J. Wearing, and Natalia L. Komarova

Contents

1 Further details of building the analytical model 2
1.1 Division tree probability calculations: an example 2
1.2 Building the equations 2

2 Further analysis of the deterministic mutant dynamics 6

3 Further details on stochastic simulations 8

4 Development 8

5 Asymmetric divisions 10

6 The role of compartment sizes 14

7 Comparison with experimental data 16

1 Further details of building the analytical model

1.1 Division tree probability calculations: an example

The process of calculating probabilities of different division trees is illustrated here. What are the possible patterns of replacement of 2^n cells? For example, if $n = 3$, we have 5 possibilities to replace 2^3 cells as illustrated in Fig A. Fig A(a) corresponds to 4 proliferation events in compartment C_2 (4 differentiation chains of length one). Fig A(b,c) represent one differentiation chain of length 2 and two differentiation chains of length 1. Fig A(d) corresponds to 2 differentiation chains of length 2. In the last pattern, Fig A(e), we have one differentiation chain of length 3.

1.2 Building the equations

Here we illustrate the process of calculating components of the ODEs described in the main text. A program in *Mathematica* has been written to generate analytical expressions for the division trees and their probabilities, and to formulate the ODEs describing mutant dynamics. Fig B presents the example of $n = 4$ (i.e. 5 compartments in the system). The first column lists all the division trees that have a nonzero probability. Each tree is represented as a 5-component vector, where the top entry by construction is 2^n and corresponds to the number of cells removed from compartment C_n, and each subsequent entry is the number of differentiation events in each of the upstream compartments. For example, the first vector $(16, 8, 0, 0, 0)^T$ corresponds to 8 differentiation divisions in compartment C_3 followed by 8 self-renewal events in the same compartment, such that no additional differentiations in the upstream compartments take place. Note that in Fig B differentiation trees are denotes as column vectors, which is different from the form of Eq (1) of the main text. The last vector in the "Division tree" column of Fig B, $(16, 8, 4, 2, 1)^T$, is a chain of differentiations in all the compartments which ends in a differentiation from the stem cell compartment, C_0, and a self-renewal division in the same compartment. Note that the self-renewal divisions are not explicitly listed.

The second column in Fig B resents the total change in the number of mutants in each of the compartments, corresponding to each division tree.

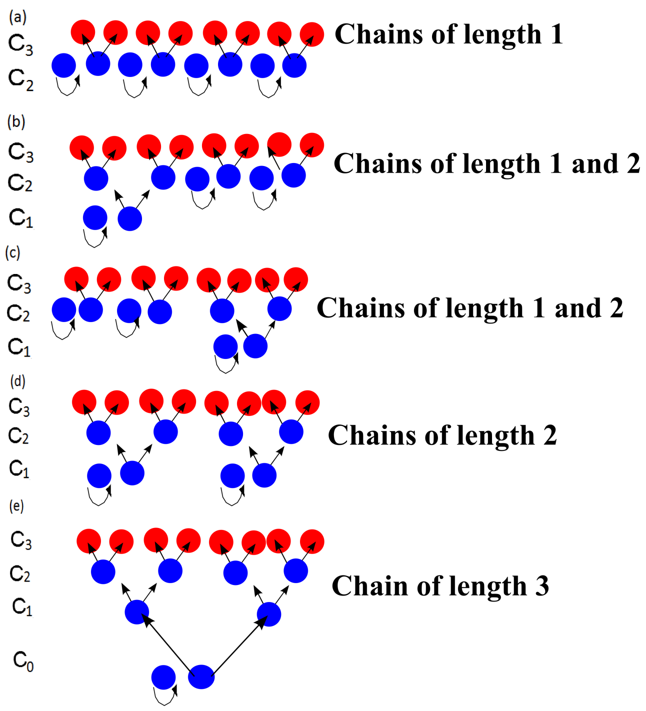

Figure A: Possible patterns to replace $2^3 = 8$ cells removed from compartment C_3. The cells in red correspond to 8 cells discarded in compartment C_3 and the blue cells are dividing cells (again, only dividing cells are shown, and the actual compartments contain more cells). (a) All 4 cells that differentiated out to replace 8 cells in C_3 are replaced by 4 proliferation events in compartment C_2. This pattern occurs with probability v^4. (b) and (c): 2 out of 4 cells that differentiated out from compartment C_2 are replaced by one differentiation event from compartment C_1 (followed by a proliferation in C_1) and two cells in C_2 are replaced by 2 proliferation events in compartment C_2. The probability of each of these patterns is $v^2(1-v)^2$. (d) The 4 cells that differentiated out from compartment C_2 are replaced by 2 differentiations from compartment C_1, followed by 2 proliferation events in C_2. The probability of this pattern is $(1-v)^4v^2$. (e) Similar to (d), except that the 2 cells in C_1 are replaced by a differentiation from C_0 followed by a proliferation event. This pattern occurs with probability $(1-v)^6$.

This is calculated in the absence of new mutations, and the notation

$$\Delta_i = \frac{m_i}{N_i} - \frac{m_{i+1}}{N_{i+1}}$$

is used. The third column is the probability of each of the division trees, which are functions of the self-renewal probabilities in the compartments.

In order to construct the ODEs governing mutant dynamics in the absence

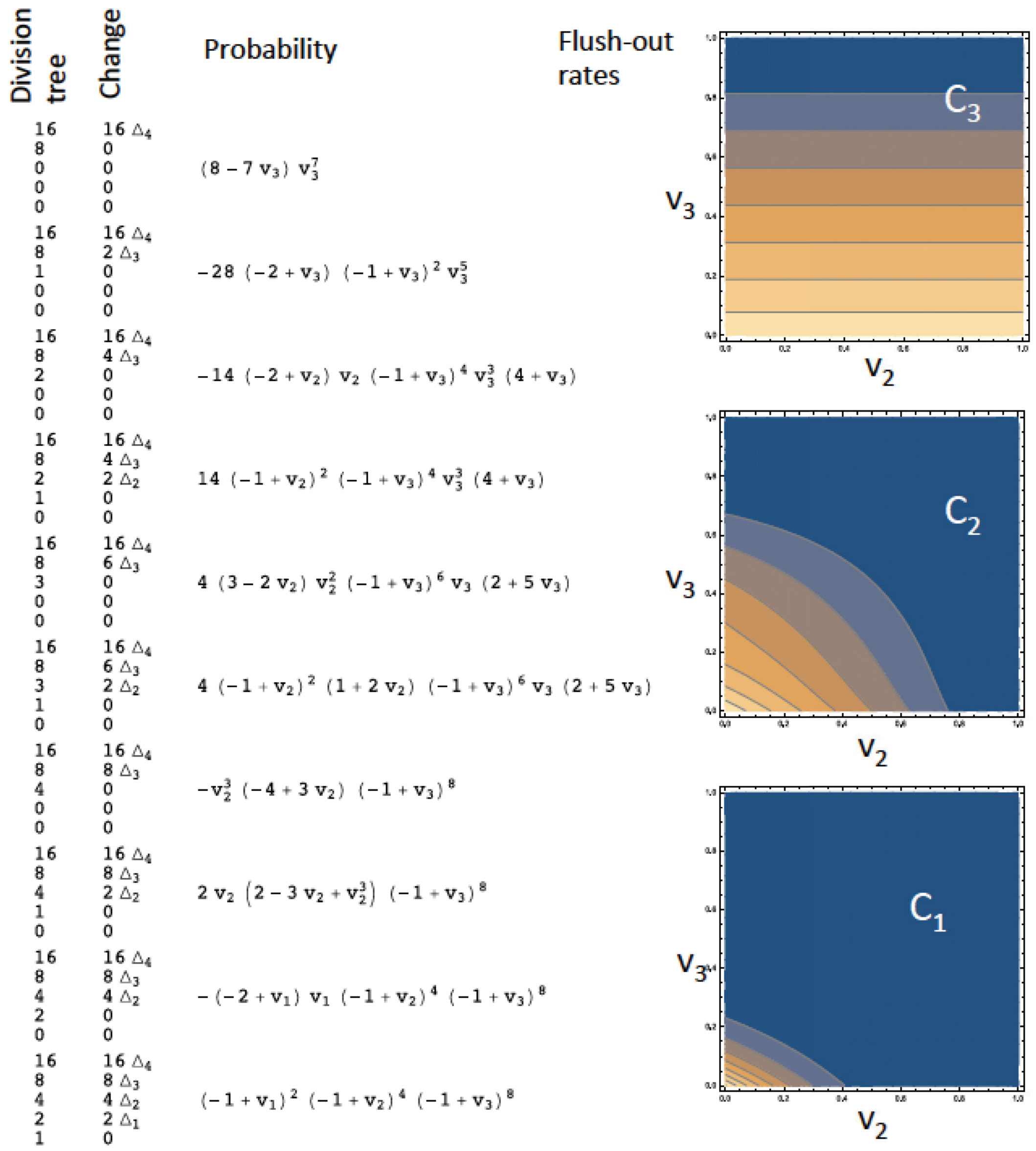

Figure B: Components of the analytical model for $n = 4$ (5 compartments). The 3 columns on the left list the division trees, the associated change in the number of mutants, and the probability of each tree. The rightmost column shows heat plots of the flush-out rates for each compartment, $K_1^{(v)}$, $K_2^{(v)}$, and $K_3^{(v)}$, as functions of v_2 and v_3, with $v_1 = 0.1$.

of new mutations, one simply adds all the "Change" vectors multiplied with their probabilities. The resulting vector of length $n+1$ is the right hand side of the mutant dynamics ODEs, system (7) of the main text.

One can see that the right hand side of the equation for $\dot{m}_i$ multiplies Δ_i. The multipliers, which we termed "flush-out rates", $K_i^{(v)}$, are functions of the self-renewal rates v_i. It follows that the flush-out rate for compartment C_0 is zero, as explained in the main text. The flush-out rate from compartment C_1 is obtained from only one tree (the longest one) and thus is proportional to the probability of that tree, which is a function of v_1, v_2, and v_3. The change in each of the downstream compartments is a function of a subset of self-renewal probabilities, with the change in the last compartment being a function of v_3 only.

For general values of n, the following patterns hold:

- $K_1^{(v)} = 2\prod_{i=1}^{n-1}(1-v_i)^{2^i}$. Eq (8) of the main text follows if $v_i = v$.
- The flush-out rate in compartment i is a function of $v_i, \ldots, v_{n-1}$. In particular, $K_{n-1}^{(v)}$ is a function of v_{n-1} only.
- For the most differentiated compartment, $K_n^{(v)} = 2^n$ is independent of v_i.
- For the special case where $v_i = 0$, that is, cells do not proliferate in any compartment except for C_0 we have $K_i^{(v)} = 2^i$, and we obtain the following system:

$$\dot{m}_0 = 0, \quad (1)$$

$$\dot{m}_1 = 2\left(\frac{m_0}{N_0} - \frac{m_1}{N_1}\right), \quad (2)$$

$$\dot{m}_2 = 4\left(\frac{m_1}{N_1} - \frac{m_2}{N_2}\right), \quad (3)$$

$$\dot{m}_3 = 8\left(\frac{m_2}{N_2} - \frac{m_3}{N_3}\right), \quad (4)$$

$$\ldots$$

- The flush-out rates are non-increasing functions of the self-renewal probabilities, v_i.

The flush-out rates in each of the compartments are presented in the last column of Fig B. There, the heat plots of the rates $K_1^{(v)}, K_2^{(v)}$, and $K_3^{(v)}$ are shown as functions of v_2 and v_3. We can see that $K_3^{(v)}$ only depends on v_3. Rate $K_2^{(v)}$ is a function of v_2 and v_3. Rate $K_1^{(v)}$ is a function of three variables, v_1, v_2, and v_3, and for the graph in Fig B we kept $v_1 = 0.1$. The graphs illustrate the non-decreasing dependence of the flush-out rates on their variables, v_i.

It is also instructive to study the probability of mutant generation in the stem compartment. The equation for $\dot{m}_0$, for a general system size n, is given by

$$\dot{m}_0 = 2u \prod_{i=1}^{n-1} (1 - v_i)^{2^i} \left(1 - \frac{m_0}{N_0}\right).$$

In the absence of pre-existing mutations, the rate of new mutation generation in compartment C_0 is given by $2u \prod_{m=1}^{n-1} (1 - v_i)^{2^i}$ and thus it is a monotonically decreasing function of rates v_i.

2 Further analysis of the deterministic mutant dynamics

Here we provide some analysis of the large mutation rate, large population regime ($uN \gg 1$). The ODE approximation can be generated with the *Mathematica* program. Equations for $n = 1$, $n = 2$, and $n = 3$ are presented in the main text. For $n = 4$ we have

$$\begin{aligned}
\dot{m}_4 &= 16(u + \mu_3(1-u) - \mu_4), \\
\dot{m}_3 &= -8 \left(u \left(16(\mu_2 - 2\mu_3 + 1)v^8 - 64(\mu_2 - 2\mu_3 + 1)v^7 + 112(\mu_2 - 2\mu_3 + 1)v^6\right.\right. \\
&- 112(\mu_2 - 2\mu_3 + 1)v^5 + 70(\mu_2 - 2\mu_3 + 1)v^4 - 28(\mu_2 - 2\mu_3 + 1)v^3 + 7(\mu_2 - 2\mu_3 + 1)v^2 \\
&- \left.\left. 2(\mu_2 - 2\mu_3 + 1)v + \mu_2 - 1\right) - (\mu_2 - \mu_3)(v-1)^2 \left(16v^6 - 32v^5 + 32v^4 - 16v^3 + 6v^2 + 1\right)\right), \\
\dot{m}_2 &= 4(v-1)^2 \left((\mu_1 - \mu_2)(v-1)^4 \left(2v^6 + 12v^5 - 2v^4 + 4v^3 + 18v^2 + 1\right) - u \left(2(\mu_1 - 2\mu_2 + 1)v^{10}\right.\right. \\
&+ 4(\mu_1 - 2\mu_2 + 1)v^9 - 38(\mu_1 - 2\mu_2 + 1)v^8 + 76(\mu_1 - 2\mu_2 + 1)v^7 - 8(7\mu_1 - 18\mu_2 + 11)v^6 \\
&- 4(7\mu_1 + 2\mu_2 - 9)v^5 + (91\mu_1 - 118\mu_2 + 27)v^4 - 8(9\mu_1 - 14\mu_2 + 5)v^3 + 12(2\mu_1 - 3\mu_2 + 1)v^2 \\
&- \left.\left. 4(\mu_1 - 2\mu_2 + 1)v + \mu_1 - 1\right)\right), \\
\dot{m}_1 &= 2(v-1)^6 \left(-14(\mu_2 - 1)uv^3(v+4) + (v-1)^8(-\mu_0 u + \mu_1 - \mu_1 + u) + 2(\mu_1 - 1)u(v-2)v(v-1)^6\right. \\
&- \left. 2(\mu_1 - 1)uv(v+2)(v-1)^4 - 4(\mu_1 - 1)uv(2v+1)(5v+2)(v-1)^2\right), \\
\dot{m}_0 &= 2u(v-1)^{14}(1 - \mu_0).
\end{aligned}$$

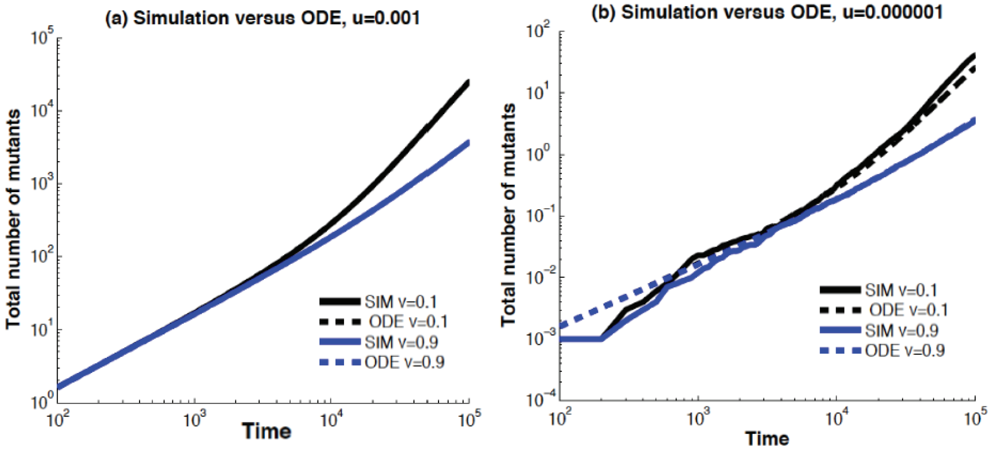

Figure C: Comparison between the average number of mutants from 1000 stochastic simulations and the number of mutants predicted by the ODE approximation, for low ($v = 0.1$) and high ($v = 0.9$) values of the self-renewal probability. (a) $u = 10^{-3}$, (b) $u = 10^{-6}$. Solid lines correspond to the deterministic and dashed lines to the stochastic calculations. We use $n = 3$ and the compartment sizes are $N_0 = 10^3, N_1 = 10^4, N_2 = 10^5, N_3 = 10^6$.

The stochastic and deterministic models coincide in the regime where $uN \gg 1$, as demonstrated in Fig 3 of the main text and further in Fig C, where in panel (b) we used lower mutation rates.

Fig D shows details of mutant accumulation dynamics for two opposing tissue architectures: increasing compartmental sizes (blue) and decreasing compartmental sizes (green). One can see that at first, the linear growth of mutants in each compartment is the same for both models; the stem cell compartment has the smallest number of mutants while the mature compartment has the largest because of the larger number of divisions in that compartment. Later on, however, differences in the behavior of the two architecture types appear. In the system where the compartment size increases from the stem cell compartment to the mature cell compartment (blue), the smallest compartment is the most "protected" (has fewer divisions), and the largest compartment accumulates mutations quickly, therefore such a system is relatively efficient in mutation accumulation. In the opposite case (green), the largest number of mutations has to accumulate in the most protected (stem cell) compartment, and this naturally takes longer. While at the steady state, both systems have the same number of mutations, the transient behavior is

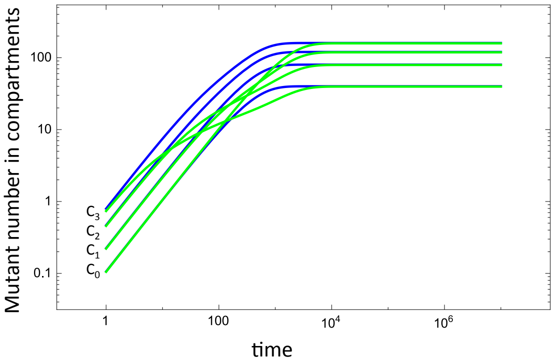

Figure D: The ODE mutant dynamics for 4 compartments, under two architectures. Blue lines correspond to increasing compartment size: $N_0 = 40, N_1 = 80, N_2 = 120, N_3 = 160$, and green lines to the decreasing compartment sizes, $N_0 = 160, N_1 = 120, N_2 = 80, N_3 = 40$. The compartments are labeled $C_0, \ldots, C_3$ to the left of the lines. The other parameters are $v = 0.1$ and $u = 0.1$.

characterized by a smaller number of mutations in the architecture where compartment size decreases from C_0 to C_n.

3 Further details on stochastic simulations

In Fig 5 of the main text and also in similar figures of this supplement we present the mean results of stochastic simulations. Fig E provides information on the underlying distribution of the numbers of mutants. The histograms of the numbers of mutations obtained among 1000 independent simulations are presented for $10 = 10^2$ (blue) and $t = 10^5$ (orange). A histogram of the log transformed data is shown in Fig E(b).

4 Development

In the main text, adult tissue at homeostasis is considered, and the optimization task is to minimize dangerous mutations as time advances. This is strictly not correct, because we set zero mutants as the initial condition,

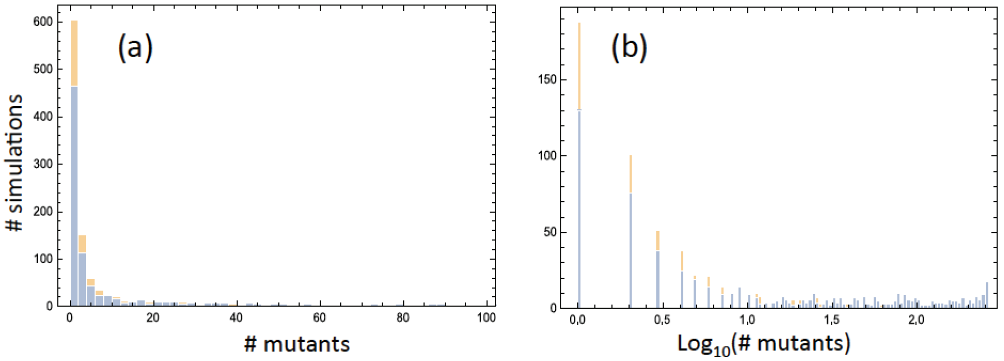

Figure E: Typical histograms of the numbers of mutants in 1000 independent stochastic simulations for $10 = 10^2$ (blue) and $t = 10^5$ (orange). (a) Linear scale, (b) Logarithmic scale. The parameters correspond to one of the cases presented in figure 5 of the main text : $n = 3$, $N_0 = 65, N_1 = 65, N_2 = 65, N_3 = 65$, $v = 0.1$, $u = 10^{-3}$.

while it is possible that mutations can be generated during development of the organ.

There are multiple ways to implement the developmental stage. It is possible that during development, cellular decisions are determined differently, and cellular signaling is orchestrated differently, due to some switches that distinguish between the developmental stage and homeostasis. The mouse literature, for example, provides evidence that the epidermis develops in stages with different division patterns, such that primordial epidermal cells divide strictly symmetrically, and after a while, they switch to a mixed mode, consisting of both symmetric and asymmetric divisions Poulson and Lechler (2010); Ray and Lechler (2011); Poulson and Lechler (2012).

Here we have implemented one way of "growing the tissue" that does not include qualitative or quantitative differences in decision making during development and homeostasis. We start with only the stem cell compartment (compartment C_0) at full capacity, and all other compartments empty. To fill compartment C_1, we treat the tissue as a 2-layer system which has had all "terminal" cells die; then we probabilistically determine how to replenish the missing cells by divisions from the 0th compartment. To fill compartment C_2, we treat the tissue as a 3-layer system which has had all "terminal" cells die, and probabilistically determine how to replenish the missing cells with the lower layers; etc. Allowing mutants to form during this process

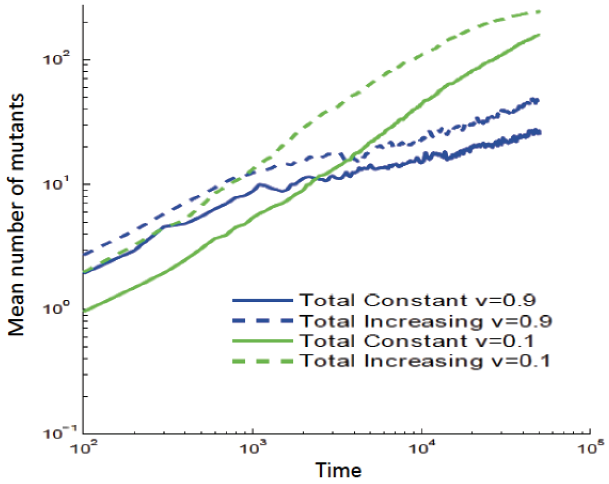

Figure F: The mean number of one-hit mutants observed in stochastic simulations that include the developmental stage. Parameters are as in Fig. 5(c) of the main text.

provides one an opportunity to study how mutant cells in tissue development accumulate. Results of the simulations that include the developmental stage are presented in Fig F for the mean number of one-hit mutants, and in Fig G for the time distribution until the generation of two-hit mutants. We can see that the results are qualitatively similar to those obtained in the absence of the developmental stage. This is consistent with the results of Derényi and Szöllősi (2017), who showed that in their optimization problem, the developmental stage only resulted in a small correction.

5 Asymmetric divisions

In the main text, only symmetric divisions (self-renewals and differentiations) are included. While it has been argued that in colonic crypts, most divisions are symmetric Lopez-Garcia et al. (2010); Snippert et al. (2010); Simons and Clevers (2011); Klein and Simons (2011), other tissues may have varying percentages of asymmetric divisions. For example, the mouse epidermis is characterized by about 80% asymmetric divisions in the ear and tail tissue, and about 60% symmetric divisions in the paw tissue Clayton et al. (2007); Doupé et al. (2010); Mascré et al. (2012); Lim et al. (2013). Here we study

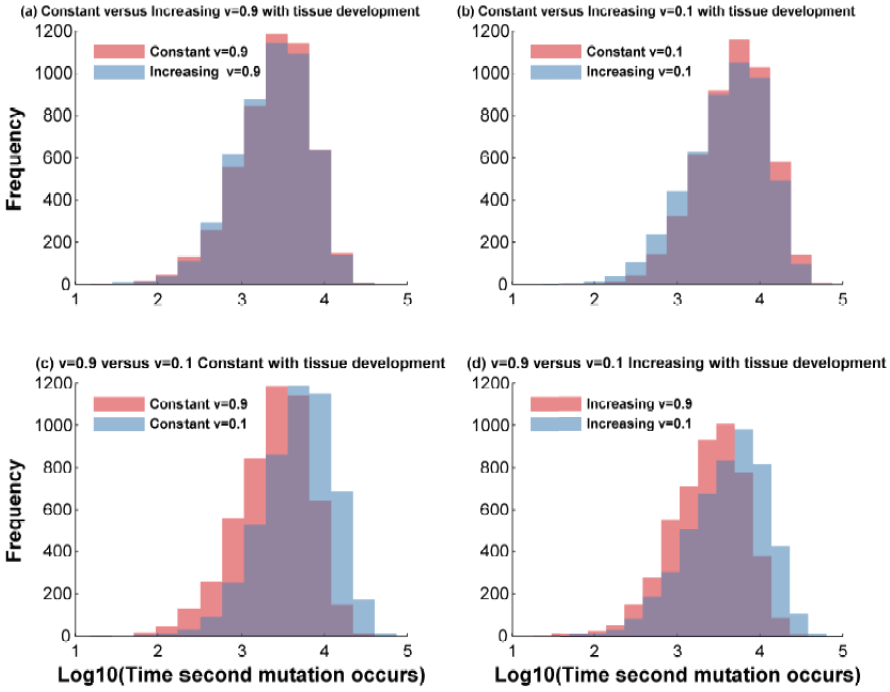

Figure G: The time to two-hit mutant generation obtained in stochastic simulations that include the developmental stage. Parameters are as in Fig 6 of the main text.

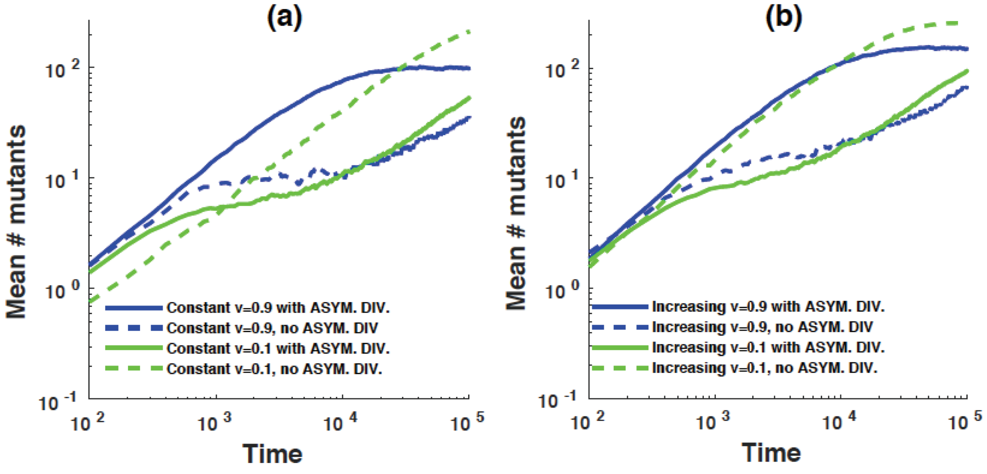

Figure H: The mean number of one-hit mutants observed in stochastic simulations that include asymmetric divisions at 90%. The rest of the parameters are as in Fig 5(c) of the main text.

the consequences of adding asymmetric divisions. To exaggerate the effect, we included asymmetric divisions at a high frequency of 90%.

To include asymmetric divisions, we implemented the following modification to the algorithm of the main text: every time there is a differentiation event in compartment C_{i-1} to replenish cells in compartment C_i, with probability $\sigma = 0.9$, an asymmetric division was implemented instead of a symmetric differentiation event. For each asymmetric division, with probability $u/2$ a mutant is produced and stays in compartment C_{i-1}, and with probability $u/2$ a mutant enters compartment C_i.

Typical simulation results are presented in Fig H for the mean number of one-hit mutants, and in Fig I for the distribution of time until the generation of two-hit mutants. We observe that adding a significant proportion of asymmetric divisions changes the dynamics of one-hit mutants (Fig H). The most striking effect that we observe when we compare similar systems with and without asymmetric divisions (see also Fig 5(c) of the main text), is that the $v = 0.1$ and $v = 0.9$ lines seem swapped in the simulations with asymmetric divisions.

Let us examine the behavior of small-v and large-v systems more closely. In the absence of asymmetric divisions, small values of v lead to a larger number of mutants, due to a larger probability of creating mutations in com-

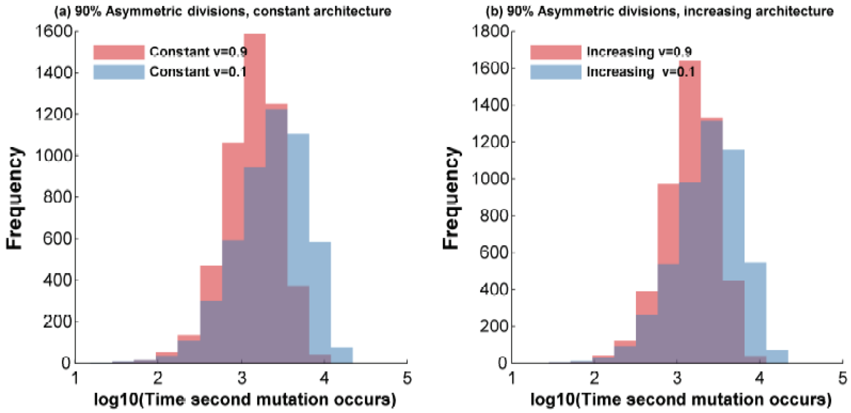

Figure I: The time to two-hit mutant generation obtained in stochastic simulations that include asymmetric divisions at 90%. The rest of the parameters are as in Fig 5(c) of the main text.

partment C_0, which can fixate in the population. This feature of small-v systems becomes relatively less important in the presence of asymmetric divisions. Indeed, when an asymmetric division occurs in compartment C_{i-1}, no cells are removed from that compartment, which reduces the chance of having long chains of differentiations. As a consequence, we observe a relatively small number of mutants in the stem cell compartment. In other words, small-v systems continue to have the advantage of fast flush-out rates of mutants, but they no longer have the disadvantage of creating mutants in the stem cell compartment. Therefore, systems with small v values in the presence of asymmetric divisions are characterized by a smaller number of one-hit mutants, compared to systems with large values of v. This is what we observe in Fig H, when we compare solid blue and green lines. The blue lines correspond to $v = 0.9$, and they indicate more mutants compared to the green lines ($v = 0.1$). Small-v systems continue to produce two-hit mutants slower than large-v systems; the presence of asymmetric divisions does not reverse this trend.

In Fig I again we used 90% asymmetric divisions. We observe clearly that $v = 0.1$ is by far a better option than $v = 0.9$ in that the production of two-hit mutants is significantly delayed in the case of small values of self-renewal probability. In panel (a) the mean values for $\log_{10}$ of waiting time are 3.1243 and 3.3520 for $v = 0.9$ and $v = 0.1$, respectively. For panel (b), the mean

values are 3.1558 and 3.3689 for $v = 0.9$ and $v = 0.1$, respectively. The p values are very very small for both panels ($p \ll 0.05$).

6 The role of compartment sizes

The question of optimal tissue architecture can be addressed in many different ways. If the total number of cells and the number of compartments are fixed, there is a very large number of ways to arrange individual compartment sizes. It is a difficult problem to find the optimum among all possible compartment size arrangements. At the same time, we know that the size distribution of compartments is determined by more than one factor. For example, it would be unrealistic to say that the number of cells in the SC compartment is large and the number of terminally differentiated cells is small, because it is the more mature compartments that are there to perform the function of the cells, and from the functional perspective, it is the numbers of such cells that have to be maximized.

To study the question of mutant generation and the role of the compartment sizes, we will not attempt to consider all possible compartment size arrangements. Instead, we will look for general trends. From Eq (7) in the main text we can see that to increase the outflow of mutants from each compartment, one should decrease the N_k/N_{k-1} ratio. Basically, the inflow and the outflow of mutants are both proportional to their concentrations, and to increase the relative size of the outflow one should increase the concentration in compartment C_k and decrease the concentration in compartment C_{k-1}.

This simple argument provides an important constraint on mutant production and dynamics. To study this constraint, we will consider three types of tissue architecture: increasing compartment sizes, constant compartment sizes, and decreasing compartment sizes. The latter case is clearly unrealistic, and is included for completeness.

In the simplest case, we fix the probability of proliferation to be constant among the compartments (for $0 < i < n$), and compare the expected mutant production following a (rare) mutation event for different arrangements of compartment sizes. Fig J illustrates three different types of architecture: exponentially increasing from N_0 to N_n (blue lines), constant (green lines) and exponentially decreasing from N_0 to N_n (yellow lines). The two panels show the expected number of mutants on different time scales. In Fig J(a), on the time scale of this plot, for small v, decreasing compartment size minimizes

mutations, and for large v, increasing size minimizes mutations. In Fig J(b), the same plot is shown for longer time scales. Now decreasing compartment size minimizes mutations for both values of v.

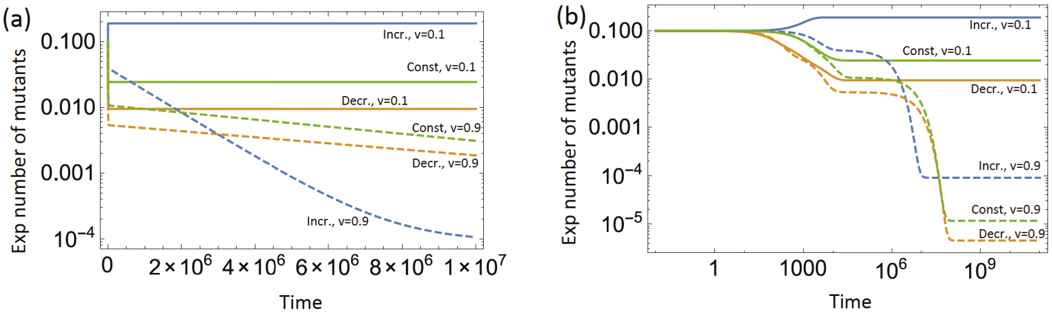

Figure J: Comparison of mutant dynamics for different compartment size arrangements. The expected number of mutants is shown as a function of time for increasing compartment size $N_i = 80e^{i+1}$ (blue lines), constant compartment size (green lines), and decreasing compartment size $N_i = 80e^{5-i}$ (yellow lines), for $0 \leq i \leq n = 4$. The total population size is the same for all systems. The rare mutation limit is assumed. The probability of proliferation in each compartment is constant, $v_i = v$ for $0 < i < n$, and two values of v are used: $v = 0.1$ (solid lines) and $v = 0.9$ (dashed lines). (a) Shorter time-scales. (b) Longer time-scales (note the log scale on the horizontal axis).

In the model studied so far, v and the compartment sizes are assumed independent, which might be an over-simplification. Therefore, we explore a model where the proliferation probability v is a function of compartment sizes. Suppose that a number of cells differentiated out of the C_k compartment. Then compartments C_k and C_{k-1} might "compete" to fill the empty spaces. It is possible that compartment C_k is small and C_{k-1} is large (and crowded?). In this case, differentiations from C_{k-1} to C_k are preferred, and the probability of proliferation, v_k, is relatively low. One possibility is to set $v_k = N_k/(N_k + N_{k-1})$, or, more generally,

$$v_0 = 1, \quad v_k = \frac{N_k^\beta}{N_k^\beta + N_{k-1}^\beta}, \quad 1 \leq k \leq n-1, \quad v_n = 0. \tag{5}$$

Again, the optimization task is to arrange the populations in the different compartments to minimize mutation production. Fig K illustrates some of

the patterns we have observed. It compares two opposite arrangements of compartment sizes, increasing from N_0 to N_n (blue lines), and decreasing from N_0 to N_n (yellow lines). In the example provided, the increase or decrease is exponential, but other laws have also been investigated and give qualitatively similar results. According to Eq (5), the increasing compartment architecture leads to higher values of v than the decreasing architecture.

Consider an extreme scenario where $\beta \to \infty$ in Eq (5). If the compartment size grows from C_0 to C_n, we have $v_k = 1$ for $0 \leq k \leq n-1$, and only trees of length 1 exist. If the compartment size decreases from C_0 to C_n, we have $v_k = 0$ for $1 \leq k \leq n$, and all the trees have length n.

For increasing compartment sizes, mutants can only appear in compartment C_{n-1}, and they fixate with probability m_0/N_{n-1} (where m_0 is the initial number of mutants), such that the expected colony size is given by m_0. For decreasing sizes, mutants can appear in all compartments, but they are flushed out from any compartment except from C_0, where they fixate with probability $1/N_0$. So, only mutants that originate in C_0 can give rise to fixation in the state

$$\frac{m_0}{N_0}\sum_{k=0}^{n-1} N_k. \tag{6}$$

The probability that a mutation occurs in compartment C_0 (given that a mutation occurs) is

$$\frac{1}{1+\sum_{i=0}^{n-2} 2^i} = 2^{1-n}. \tag{7}$$

The product of factors Eq (6) and Eq (7) defines the long time behavior of the mutant numbers for the decreasing compartment sizes in Fig K, as β increases. In the limit where $\beta \to \infty$, the increasing compartment system is characterized by a constant (and equal to m_0) number of mutants.

7 Comparison with experimental data

We used the experimental data obtained by Buske et al. (2011) on the positional BrdU label index, which is an indication of cellular division activity. The data from two measurements presented in Buske et al. (2011) are plotted in Fig L(a), small circles. Position 0 is the nearest to the stem cells. We

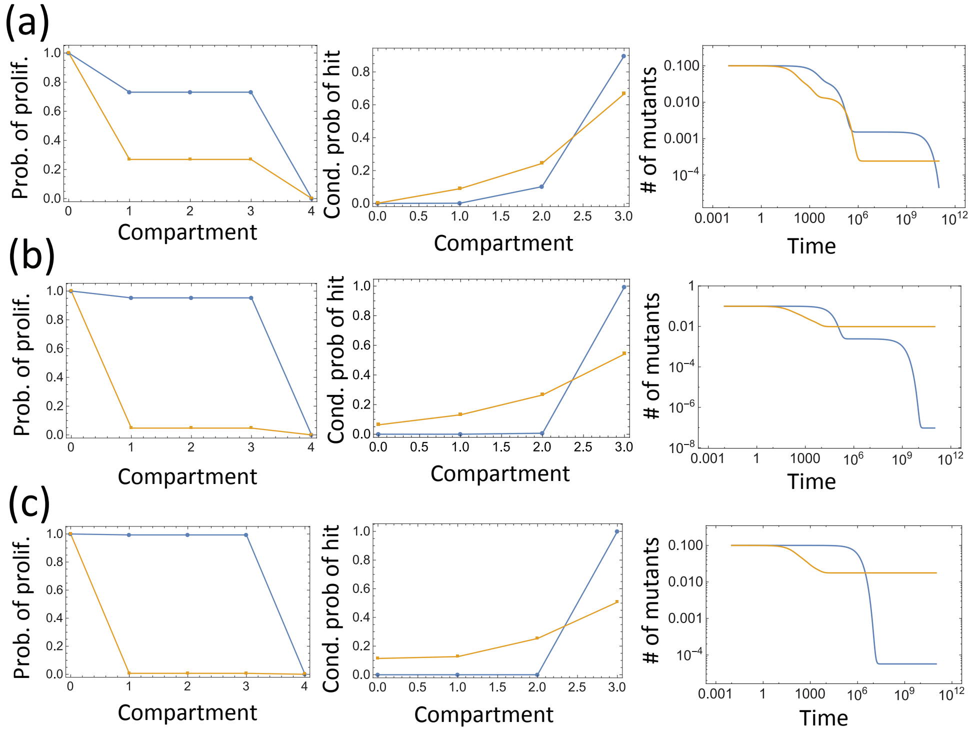

Figure K: Comparison of mutant dynamics for two different compartment size arrangements: increasing compartment size $N_i = 80e^{i+1}$ (blue lines), and decreasing compartment size $N_i = 80e^{5-i}$ (yellow lines), for $0 \leq i \leq n = 4$, $m_0 = 0.1$. The rare mutation limit is assumed. The probability of proliferation in each compartment, v_k, is determined by Eq (5), where (a) $\beta = 1$, (b) $\beta = 3$, and (c) $\beta = 5$. The values of v_k are shown in the leftmost plots. The conditional probability that a mutant is generated in each compartment (given that a mutant is generated) is plotted in the middle graphs. The rightmost graphs show the relative number of mutants in both systems as functions of time. Note that these graphs can only be interpreted for purposes of comparison, not as absolute numbers.

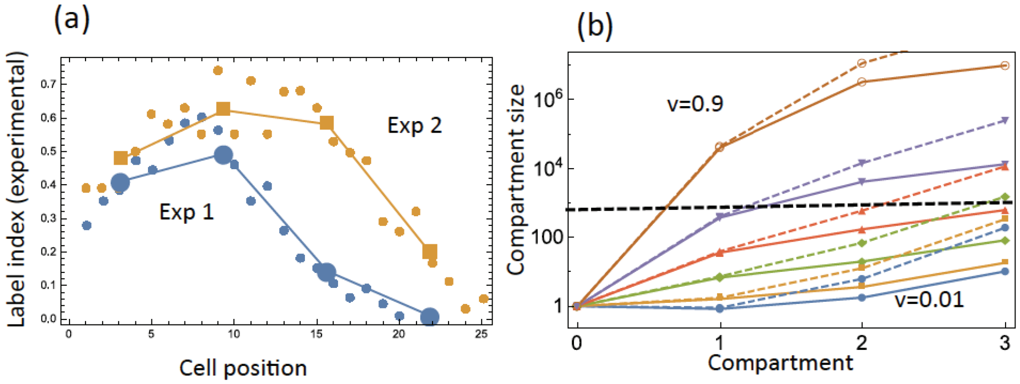

Figure L: Comparison of theory and experimental data. (a) The positional BrdU label index as reported at two different time points (2 and 24 hours after labeling) in an experiment presented in Buske et al. (2011) (small circles). The mean index for $n = 3$ compartments is shown as large dots and squares. (b) The compartment size required for our theory to reproduce the experimental values (solid/dashed lines for the Exp1/Exp2 in (a), respectively). The different curves correspond to $v = 0.01, 0.1, 0.3, 0.5, 0.7, 0.9$ from bottom to top. The dashed horizontal line is an approximate cut-off for the sizes.

would like to see under which values of v this information can be reproduced with our model.

First, we will assume $n = 3$ for colonic crypts and calculate the value of the label index for each compartment by splitting the data points of Fig L into $n+1$ groups by location and calculating the mean index for each group. Let us denote the resulting vector of mean label index as

$$\{B_0, \ldots, B_n\}.$$

Such vectors are plotted in Fig L(a) with large circles and squares.

Next we would like to see if our model can produce expected numbers of divisions that match these values. For a given division tree (Eq (1) of the main text),

$$\{a_0, \ldots, a_{n-1}\},$$

the entries denote the number of differentiation divisions in each compartment. We can calculate the total number of divisions in each compartment, c_i, as

$$c_i = a_i + (a_i - 2a_{i-1}), \quad 1 \le i \le n-1, \quad c_0 = a_0 + a_0$$

where the first term is the number of differentiation divisions and the 2nd term the number of self-renewal divisions in compartment C_i. Given the probability of each tree, we can calculate the expected number of cell divisions in compartment i, b_i, as a weighted sum of all the values c_i corresponding to different division trees:

$$b_i = \sum_{\text{All trees}} \text{Prob}^{\text{tree}} c_i^{\text{tree}}.$$

The values b_i are functions of the self-renewal probabilities, v_i. For the purposes of this section we will take constant values $v_i = v$. Let us denote the vector of the predicted numbers of divisions by

$$\{b_0, \ldots, b_n\}.$$

For both the vector of mean label index and the vector of the mean numbers of divisions, only the relative values are important, so we can normalize these vectors to sum up to 1. In order to create the predicted index values (which are the values averaged over the compartment), we set

$$B_i = b_i/N_i, \tag{8}$$

where on the left we have the experimentally obtained values, and on the right the numerator is a function of v, and the denominator is the compartment size. The question is, for a given value of v, can we find compartment sizes N_i that satisfy Eq (8)in S1 Text. Fig L(b) depicts the calculated relative values of $N_0, \ldots, N_n$ (assuming $N_0 = 1$), for 6 different values of v: $v = 0.01, 0.1, 0.3, 0.5, 0.7$, and 0.9 (from bottom to top lines). Results for the two experiments in Fig L(a) are shown with solid and dashed lines respectively.

What we can see is quite clear: as v grows, the size of the compartments starts growing exponentially, such that for example for $v = 0.9$, the most differentiated compartment is required to be 10^6 or even 10^7 times as large as the stem cell compartment, which is unrealistic. Only values of v up to about $v = 0.3$ give compartment sizes that are realistic. This coincides with our suggestion that relatively small v values are favored.

References

Buske, P., Galle, J., Barker, N., Aust, G., Clevers, H., and Loeffler, M. (2011) , *PLoS Comput Biol* **7(1)**, e1001045

Clayton, E., Doupé, D. P., Klein, A. M., Winton, D. J., Simons, B. D., and Jones, P. H. (2007) , *Nature* **446(7132)**, 185

Derényi, I. and Szöllősi, G. J. (2017) , *Nature Communications* **8**, 14545

Doupé, D. P., Klein, A. M., Simons, B. D., and Jones, P. H. (2010) , *Developmental cell* **18(2)**, 317

Klein, A. M. and Simons, B. D. (2011) , *Development* **138(15)**, 3103

Lim, X., Tan, S. H., Koh, W. L. C., Chau, R. M. W., Yan, K. S., Kuo, C. J., van Amerongen, R., Klein, A. M., and Nusse, R. (2013) , *Science* **342(6163)**, 1226

Lopez-Garcia, C., Klein, A. M., Simons, B. D., and Winton, D. J. (2010) , *Science* **330(6005)**, 822

Mascré, G., Dekoninck, S., Drogat, B., Youssef, K. K., Brohée, S., Sotiropoulou, P. A., Simons, B. D., and Blanpain, C. (2012) , *Nature* **489(7415)**, 257

Poulson, N. D. and Lechler, T. (2010) , *The Journal of cell biology* **191(5)**, 915

Poulson, N. D. and Lechler, T. (2012) , *International review of cell and molecular biology* **295**, 199

Ray, S. and Lechler, T. (2011) , *Cell division* **6(1)**, 12

Simons, B. D. and Clevers, H. (2011) , *Cell* **145(6)**, 851

Snippert, H. J., Van Der Flier, L. G., Sato, T., Van Es, J. H., Van Den Born, M., Kroon-Veenboer, C., Barker, N., Klein, A. M., Van Rheenen, J., Simons, B. D., et al. (2010) , *Cell* **143(1)**, 134
